# *Ex Situ* Characterization of 1T/2H MoS_2_ and Their Carbon Composites for Energy Applications, a Review

**DOI:** 10.1021/acsnano.2c08913

**Published:** 2023-03-16

**Authors:** Alexandar D. Marinov, Laura Bravo Priegue, Ami R. Shah, Thomas S. Miller, Christopher A. Howard, Gareth Hinds, Paul R. Shearing, Patrick L. Cullen, Dan J. L. Brett

**Affiliations:** † Electrochemical Innovation Laboratory (EIL), Department of Chemical Engineering, University College London (UCL), Gower Street, London WC1E 6BT, U.K.; ‡ Department of Physics & Astronomy, University College London (UCL), Gower Street, London WC1E 6BT, U.K.; § National Physical Laboratory, Hampton Road, Teddington TW11 0LW, U.K.; ∥ School of Engineering and Materials Science, Queen Mary University of London, Mile End Road, London E1 4NS, U.K.; ▲ University College London (UCL), Gower St, London WC1E 6BTU.K.

**Keywords:** text scanning, MoS_2_, energy application, LIB, SIB, battery, supercapacitor, HER, characterization, TEM

## Abstract

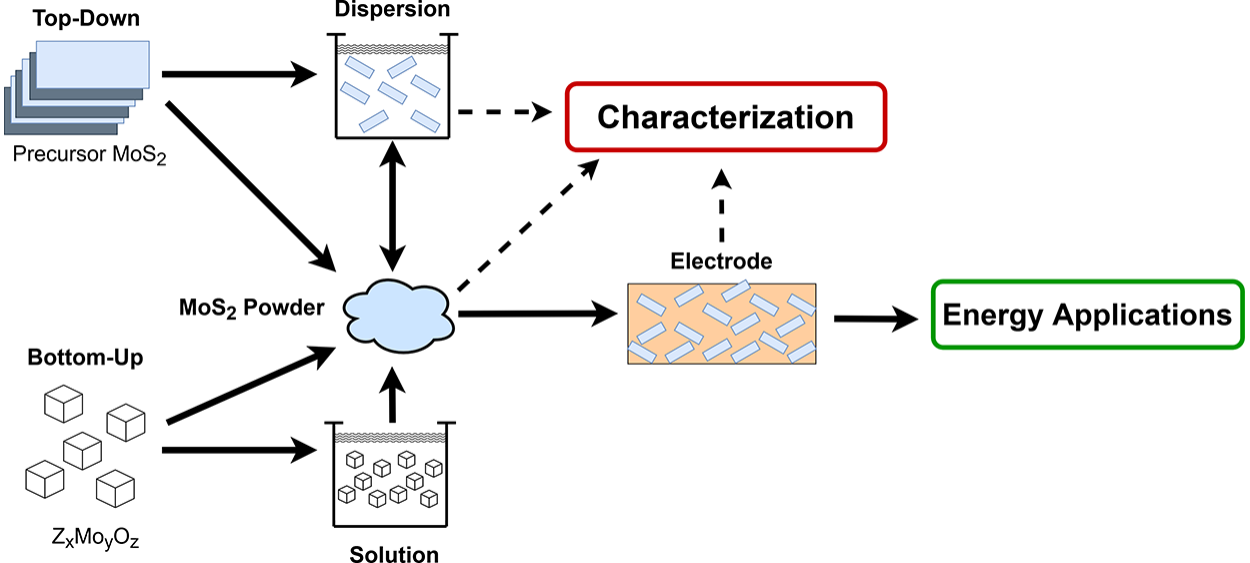

The growing interest in the development of next-generation net zero energy systems has led to the expansion of molybdenum disulfide (MoS_2_) research in this area. This activity has resulted in a wide range of manufacturing/synthesis methods, controllable morphologies, diverse carbonaceous composite structures, a multitude of applicable characterization techniques, and multiple energy applications for MoS_2_. To assess the literature trends, 37,347 MoS_2_ research articles from Web of Science were text scanned to classify articles according to energy application research and characterization techniques employed. Within the review, characterization techniques are grouped under the following categories: morphology, crystal structure, composition, and chemistry. The most common characterization techniques identified through text scanning are recommended as the base fingerprint for MoS_2_ samples. These include: scanning electron microscopy (SEM), X-ray diffraction (XRD), X-ray photoelectron spectroscopy (XPS), and Raman spectroscopy. Similarly, XPS and Raman spectroscopy are suggested for 2H or 1T MoS_2_ phase confirmation. We provide guidance on the collection and presentation of MoS_2_ characterization data. This includes how to effectively combine multiple characterization techniques, considering the sample area probed by each technique and their statistical significance, and the benefit of using reference samples. For ease of access for future experimental comparison, key numeric MoS_2_ characterization values are tabulated and major literature discrepancies or currently debated characterization disputes are highlighted.

## 1 Introduction

Developments in energy systems have attracted global attention as society transitions from fossil fuel energy sources to establish a sustainable energy future. Advancements in the fields of rechargeable batteries, supercapacitors, and electrolyzers are driving the development and expansion of energy applications. All these devices require electrodes composed predominantly of electrochemically active materials. Naturally abundant materials, which are less affected by supply chain issues and can be converted into nanostructures with enhanced material properties are at the forefront of energy research. Making these technologies economically, environmentally, and commercially viable requires the realization of low-cost large-scale production of active materials, minimal environmental impact during device manufacturing, extended device life cycles, ecologically benign material disposal, and challenging application performance targets.

**Figure 1:**
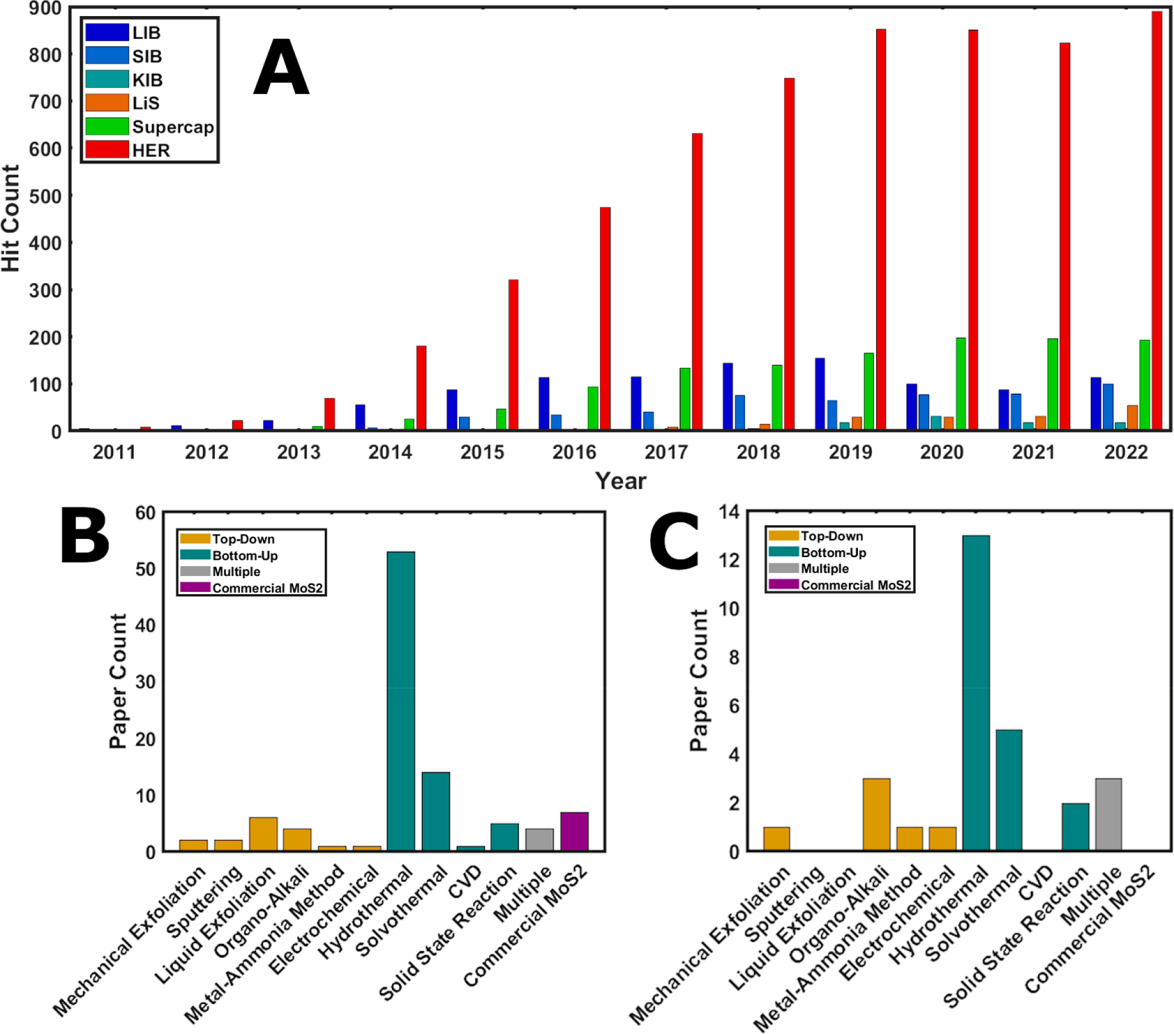
(A) Distribution of scan hits per relevant energy application category for 2011–2022 using Web of Science API (performed on 30/11/2022) and custom text classification. For detailed analysis methods, refer to the Supporting Information. Total count of 8,898 research articles presented. (B) Distribution of MoS_2_ production techniques used within the 100 manually surveyed energy application-oriented papers. (C) Production routes for 1T MoS_2_ reported by 29 out of the 100 manually surveyed energy application-oriented papers. Variance for all bar charts included in Table SI 5.

Since the experimental isolation of graphene in 2004,^1^ many other 2D materials have been successfully synthesized, a large number of which belong to the transition metal dichalcogenides (TMDs) family. TMDs are composed of group IV to XI metals covalently bonded with group XVI chalcogens, except for oxygen, in the general form MX_2_ (M = transition metal, X = S, Se, or Te). The most widely studied TMD is molybdenum disulfide (MoS_2_), whose scientific interest surged in 2013, followed by a steady increase in the number of papers published (Figures 1A and SI 1; Table SI 1).

MoS_2_ is found in the earth’s crust as the mineral molybdenite, making it economically viable to source. Alternatively, it can be produced synthetically at a higher purity from elemental molybdenum metal and sulfur. Top-down precursor MoS_2_ or commercial MoS_2_ is the nomenclature used within this review for the product of molybdenite refinement, or synthetic bulk 2H MoS_2_ production. A major benefit of MoS_2_ is that it is not known to be harmful or toxic in the top-down precursor or exfoliated form^2−4^ and can therefore be easily excavated, transported, treated, and applied. Furthermore, top-down precursor MoS_2_ has been commonly utilized in industry for many years as a catalyst or lubricant.^5^ For these reasons, the scale-up of devices involving MoS_2_ has a feasible outlook.^6−105^

Typically, review articles focus on manually bringing to light developments in a given research field. However, this omits the possibility to identify technique accessibility, method of usage, and key comparison values. Hence, in this review, 100 energy application-oriented MoS_2_ research articles including a diversity of applications, were first manually surveyed^6−105^ (Figures 1B, SI 2, and SI 3). Generally, energy application-oriented research articles include a synthesis stage, a synthesis product characterization segment, and an application testing section. Each of these is considered as a separate overarching classification category (energy application, production, and characterization) (Figure SI 4), with many sub-categories present within each overarching category. Sub-categories are first identified manually, to establish the key field terms for sub-category classification. Field terms include the field word itself and any synonym words or phrases specifically associated with that sub-category (Tables SI 2–4).

Extracting data from the literature database Web of Science using their application programming interface (API), allowed us to gather the title, abstract, and keywords text from 37,347 research articles published between 2011 and 2022. Many articles mention field terms within their title, abstract, and/or keywords. Thus, using a text scanning method (please refer to the Supporting Information API method for the full detailed methodology), it was possible to classify article energy applications and characterization techniques using the pre-established field terms. We found that a statistically significant number of MoS_2_ research publications involve energy applications (23.8%) or utilize the characterization techniques (30.1%) covered within this review. Therefore, we review the characterization technique data analyses of MoS_2_ samples for energy applications to facilitate future comparison.

### 1.1 MoS_2_ Energy Applications

Within the MoS_2_ literature, the most widely researched energy applications are hydrogen evolution reaction (HER) catalysis,^6−28^ rechargeable batteries,^29−99^ and supercapacitors^100−103^ (Figure 1A). HER is by far the most dominant research field, accounting for 66.0% of the energy applications research articles classified. For HER, the edge sites of top-down precursor MoS_2_ are inherently catalytically active, however exfoliation to nanosheets can result in the availability of additional edge sites, and even activate the nanosheet basal plane^21^ due to the existence of a conducting nanosheet phase of MoS_2_.^7−12^^,^^14,18,19,21^^,^^24−26^^,^^28^ In supercapacitors (13.5% of classified energy application research), MoS_2_ exhibits a pseudocapacitance due to the additional redox storage mechanism in combination with the double-layer capacitance.^103^

Within the field of rechargeable batteries (20.5% of classified energy application research), lithium-ion batteries (LIB)^29−74^ are the dominant research field (55.2%). However, there has been an increasing scientific interest since 2013 in emerging rechargeable batteries; sodium-ion (SIB 27.8%),^75−85^ potassium-ion (KIB 4.9%),^86−97^ and lithium sulfur batteries (LiS 9.4%).^98,99^ The wide usage of MoS_2_ across a diversity of rechargeable battery chemistries is due to the similarity between the structures of top-down precursor MoS_2_ and commercially established graphite electrodes. However, unlike graphite, which struggles with degradation due to large volume expansion when intercalating larger cations than Li^+^^97^ or is energetically unable to intercalate Na^+^ in SIBs,^76^ preferential energetics allow MoS_2_ to intercalate Li^+^,^29−74^ Na^+^,^75−85^ and K^+^^86−97^ ions.

### 1.2 Top-Down Synthesis

**Scheme 1:**
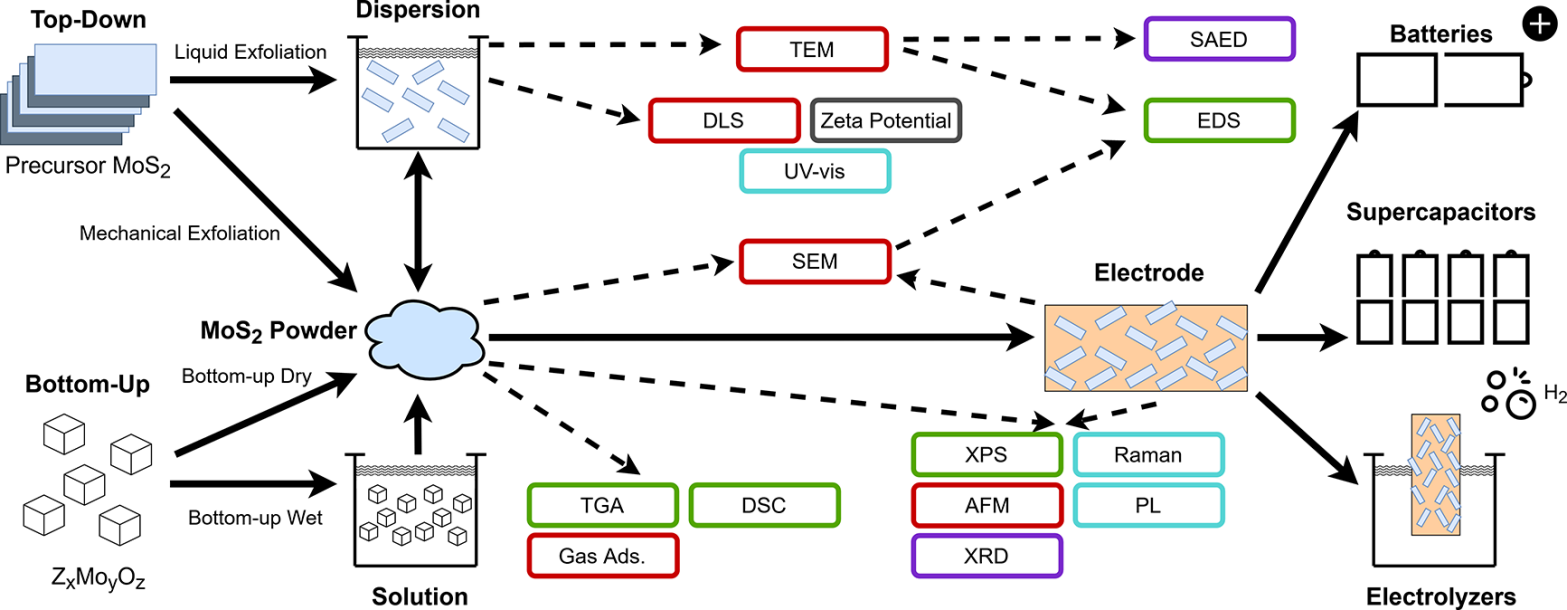
Life Cycle of MoS_2_ Samples in Research for Energy Applications Comparison of the similarities and differences in production routes, including top-down or bottom-up methods. Indication of the general similarity between sub-categories of production routes, highlighting the various stages at which characterization techniques are typically applied, followed by electrode fabrication and different energy applications. The direction of the arrows indicates the flow of processing steps. Solid arrows represent synthesis or material processing steps, and dashed arrows represent characterization steps. Color coding: morphology–red, crystal structure–purple, composition–green, chemistry–blue, and other–grey. Abbreviations: Scanning electron microscopy (SEM), transmission electron microscopy (TEM), atomic force microscopy (AFM), gas adsorption (Gas Ads.), X-ray diffraction (XRD), selected area electron diffraction (SAED), X-ray photoelectron spectroscopy (XPS), energy dispersive spectroscopy (EDS), thermogravimetric analysis (TGA), differential scanning calorimetry (DSC), photoluminescence (PL), and ultraviolet-visible spectroscopy (UV-vis).

Top-down precursor MoS_2_ is a bulk crystal layered material, where each individual MoS_2_ nanosheet consists of covalently bonded S–Mo–S layers. In the precursor, nanosheets are stacked together vertically, whereby adjacent sheets are held together by weak van der Waals forces, resulting in the precursor exhibiting flake-like morphology. The interlayer van der Waals forces allow for the top-down precursor MoS_2_ to undergo exfoliation to nanosheets, via production methods labeled as top-down synthesis^106^ (Scheme 1). Alternatively, MoS_2_ nanosheets can be grown from a variety of different reactants in bottom-up approaches (Figure 1B and C). Exfoliated and chemically grown MoS_2_ nanosheets have distinct properties relative to top-down precursor MoS_2_. These properties include increased surface area, enhanced number of available active sites, and the change from an indirect bandgap semi-conductor in the top-down precursor MoS_2_ (1.29 eV) to a direct bandgap semi-conductor (1.90 eV) in the nanosheet form.^106^

Top-down manufacture can be broadly divided into mechanical and liquid–based exfoliation routes (Scheme 1). Mechanical exfoliation relies solely on mechanical stress without solvents^1,26,86,107−110^ (Figure 1B), through techniques such as the Scotch tape method^1^ and dry ball-milling^86,108^ to induce the separation of the nanosheets, directly resulting in an exfoliated powder product (Scheme 1). On the other hand, liquid exfoliation can be further divided into pure liquid phase and intercalation-assisted liquid phase exfoliation. Commonly, liquid phase exfoliation uses both mechanical stress and a liquid environment (through sonication), to stabilize a dispersion of nanosheets from reaggregating.^6,29−31,87,100,111−115^ Alternatively, intercalation-assisted techniques including, organo-alkali solvents (*n*-butyl-lithium^7,88,102,116,117^ and Li or Na napthalenide^33,118^), the metal-ammonia method,^8,119−121^ electrochemical intercalation (Li^9^ or Na^75^), and solid state use of LiBH_4_^21^ rely on the insertion of group I alkali metal ions in between the MoS_2_ nanosheets forming the top-down precursor MoS_2_. The most widely used alkali metal intercalant is Li.

Li intercalation forms Li_*x*_MoS_2_ salt, which can be subsequently immersed in an organic, polar, aprotic solvent (such as tetrahydrofuran, THF; *N*-methyl-pyrrolidone, NMP; or *N*,*N*-dimethylformamide, DMF) in an argon atmosphere,^121^ or in deionized (DI) water in atmospheric conditions.^7−9,25,102,116,117^ In the former case, Li_*x*_MoS_2_ salt spontaneously dissolves in the solvent,^121^ while in the latter case, Li^+^ ions react vigorously with water molecules evolving hydrogen gas which violently rips the nanosheets apart.^7−9,25,102,116,117^ In both liquid exfoliation and intercalation-assisted exfoliation in water, mechanical forces such as bath or tip sonication^6,29−31,87,100,111−115^ are typically used to separate the nanosheets. In either case, the choice of a suitable solvent for the dispersion of exfoliated nanosheets slows down the restacking of nanosheets (Scheme 1). Filtering the dispersion usually results in restacked MoS_2_ powders, which can subsequently be converted into electrodes for use in energy applications.

### 1.3 Bottom-Up Synthesis

Bottom-up wet synthesis approaches include hydrothermal^10−17^,^34−59^,^76−82^,^89−92^,^98,99^^,^^103−105^,^122^ and solvothermal^18,19^,^60−64^,^83−85^^,^^89−95^^,^^123^ methods (Scheme 1 and Figure 1B). These methods include solution–based mixing of molybdenum and sulfur sources, such as Z_*x*_Mo_*y*_O_*z*_ type hydrates (*e.g.*, Na_2_Mo_2_O_4_ or (NH_4_)_6_Mo_7_O_24_) and thiourea (CH_4_N_2_S), in DI water, ethanol or DMF. The solution is subsequently treated at high temperature (approximately 200 °C) to grow various morphology MoS_2_ structures. Post synthesis, a further annealing step (approximately 700 °C) can be applied to increase the crystallinity of the structure.^10−12^^,^^15,39^^,^^48−50^^,^^54−57^^,^^76−78^^,^^99^^,^^122^

On the other hand, bottom-up dry methods include chemical vapor deposition (CVD)^65,124,125^ and solid state chemistry,^20,23,96,126−129^ which utilize solid precursors, elevated temperature and various pressure conditions to directly synthesize nanosheets or powder products (Scheme 1). In some instances, a combination of multiple production techniques can be used.^24,25,72−74,130,131^ Each production technique comes with trade-offs between production costs, both capital and operational, energy requirements associated with the manufacturing process, product morphology control, distribution of the number of nanosheet layers achieved in the final product, and the quality of nanosheets synthesized. Based on the manual survey of 100 literature articles, the most widely used production technique is the hydrothermal method (Figure 1B), which is consistent with the findings of a review article published in 2019.^132^

### 1.4 Review Aim

There already exists a plethora of high-level review articles that cover MoS_2_ nanosheet and MoS_2_ composite production routes,^106,132−135^ and their application within various devices.^106,132,136,137^ However, the characterization techniques used to validate MoS_2_ nanosheets, MoS_2_ nanosheet/carbon nanotubes (CNT), MoS_2_ nanosheet/graphene, or MoS_2_/carbon composites are only briefly mentioned in such review articles.^106,137−140^ Unlike graphene,^141^ a good practice guide on MoS_2_ characterization is not available and characterization-oriented review papers focus on specific techniques.^139,140^ Consequently, this review uses a data-driven text scanning approach to identify the most widely utilized characterization techniques applied to MoS_2_ samples, allowing for an arsenal of accessible techniques to be gathered. Within the individual techniques, this review highlights the characteristic signature expected from different MoS_2_ phases, structural forms, and their carbon composites. Furthermore, key values for characterization from 100 energy application-oriented articles are statistically analyzed and reported for ease of future comaprison. Additionally, best practices are presented throughout the review to facilitate:

- ease of comparison of MoS_2_ samples;
- reproducibility of characterization conditions;
- greater use of reference samples;
- less ambiguous classification of MoS_2_ phases;
- application of complementary characterization techniques to provide multiple sources of confirmation;
- more detailed understanding of MoS_2_ production routes and energy applications.

We thus aim to suggest a standard for the future reporting of MoS_2_ based characterization data, by using the most widely used characterization techniques as the base “fingerprint” of MoS_2_ samples.

## 2 MoS_2_ Polymorphism

MoS_2_ can exist in three different polymorphs, with its unit cell comprising of either three-layer rhombohedral (3R), two-layer hexagonal (2H), or one layer trigonal prismatic (1T).^71,127,129^ The nomenclature originates from the distinct repeating stacking order of MoS_2_ nanosheets within each polymorph. The letters A, B, and C are introduced to represent different misaligned nanosheets. Hence, in 1T phase MoS_2_ all nanosheets are aligned (AAAAAA), in 2H phase MoS_2_ alternating two layers are in alignment (ABABAB), and in 3R phase MoS_2_ alternating three layers are in alignment (ABCABC).^142^ 2H and 3R are found naturally in molybdenite but can also be produced synthetically, whereas the 1T phase is thermodynamically metastable under ambient conditions and can only be synthesized artificially. All three polymorphs appear in the literature, though 2H and 1T have been most routinely studied. HER catalysis works focus mainly on the usage of 1T due to its metallic nature,^7−12^^,^^14^^,^^18−21^^,^^24−26^^,^^28^ whereas battery work involves mainly 2H MoS_2_ because of the morphology control.^29−99^ However, some of the highest reported capacities in LIB research have been delivered by 1T MoS_2_ carbon composite structures.^33,34,37^

**Figure 2:**
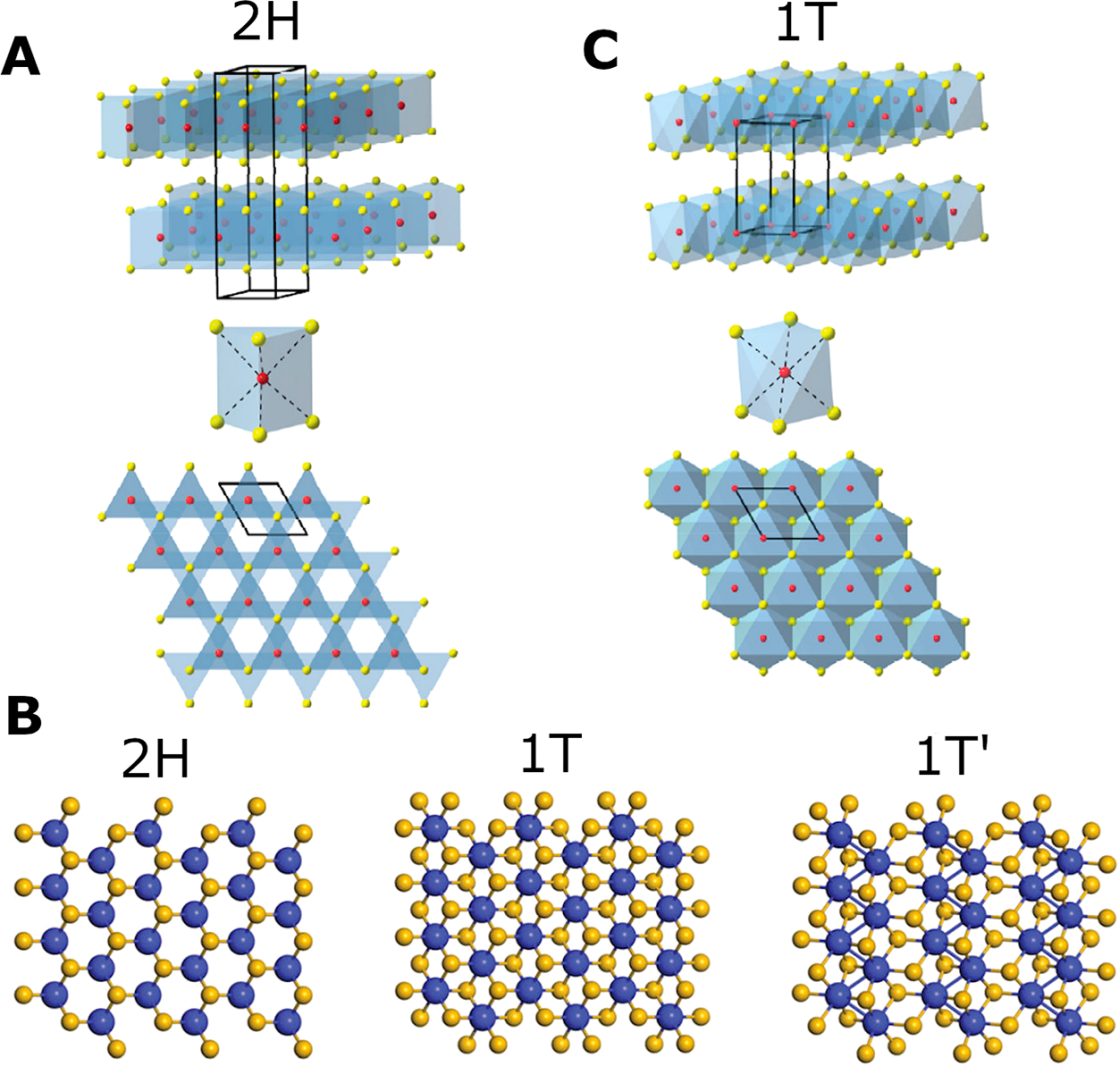
Structural representation of the polymorphs of MoS_2_. Red/blue atoms indicate Mo, and yellow atoms depict S. (A) Alignment of atoms in two adjacent layers of 2H MoS_2_, trigonal prismatic coordination of Mo atom, and top view (*c*-axis direction) of 2H nanosheet plane. (B) Top view of *c*-axis plane of the 2H, 1T, and 1T’ MoS_2_. (C) Alignment of two adjacent layers of 1T MoS_2_, octahedral coordination of Mo atom, and top view of 1T nanosheet plane. (A, C) Adapted from ref (24). Copyright 2013 American Chemical Society. (B) Adapted with permission from ref (126). Copyright 2017 Royal Society of Chemistry.

Each nanosheet of MoS_2_ is composed of S–Mo–S’ layers, whereby the S’ notation is introduced to differentiate between the top and bottom sulfur atoms in a single sheet. In 2H MoS_2_, the molybdenum and sulfur atoms misalign with their elemental counterparts in adjacent layers, resulting in the existence of a nanosheet stacking order ABABAB,^75,116^ as seen in (Figure 2A). Within a single 2H nanosheet the S and S’ atoms align vertically, resulting in S’ being hidden from view by S when viewed in the *c*-axis plane. This alignment gives rise to a hexagonal pattern observed for 2H MoS_2_ as shown in (Figure 2B), which represents a trigonal prismatic arrangement of the sulfur atoms around the central molybdenum atom.^116^

The transition from 2H to 1T can be visualized as the gliding or shift of the S’ atoms, resulting in the misalignment of S and S’ within a single nanosheet^125^ (Figure 2C). Consequently, S’ atoms protrude from underneath leading to the Mo atom appearing to be surrounded by six sulfur atoms (Figure 2B). This corresponds to an octahedral arrangement of sulfur atoms around the central Mo atom.^116^ In this case, Mo atoms between two adjacent layers are vertically aligned, leading to a nanosheet stacking order of AAAAAA in the 1T phase (Figure 2C).^75,116^ A further distorted version of the 1T polymorph exists as the 1T’ phase, which consists of a distorted octahedral structure including Mo atoms forming zigzag chains^20,126^ (Figure 2B).

It is believed that the 2H to 1T shift results from strain on the MoS_2_ lattice,^106^ commonly achieved via electron donation from intercalated Li during reductive chemistry. The 1T transition is also documented in electrochemical LIB literature.^29−74^ Only certain production routes allow for the 1T phase to be isolated (Figure 1C), these include: mechanical exfoliation methods (ball-milling^110^ and Ar gas treatment^132^), organo-alkali solvents,^7^^,^^24^^,^^25^^,^^102^^,^^116−118^ the metal-ammonia method,^8^ electrochemical intercalation,^9,75^ hydrothermal,^10−12^^,^^14^^,^^28^^,^^34^^,^^37^^,^^39^^,^^52^^,^^77^^,^^98^^,^^103^ solvothermal synthesis,^18,19,62,83,95^ and solid-state synthesis.^20,21,126,129^ Liquid exfoliation with shear stress alone cannot produce the 1T phase.^6^^,^^29−31^^,^^87^^,^^100^^,^^111−115^ Only when liquid exfoliation is coupled with another technique such as supercritical CO_2_^26^ can the phase change be achieved. *In situ* scanning transmission electron microscopy (STEM) can induce the phase change in Re-doped MoS_2_ via the electron beam, whereas Re doping alone is deemed insufficient to cause the transition.^125^

The 2H phase is semi-conducting, both in the top-down precursor MoS_2_ form and as exfoliated nanosheets. However, 2H top-down precursor MoS_2_ has an indirect bandgap of 1.29 eV and exfoliated single layer 2H MoS_2_ has a direct bandgap of 1.9 eV.^106,144^ Exfoliated 1T’ nanosheets are also considered to be semi-conducting^65^ although this is disputed,^20^ whereas 1T nanosheets are metallic, and therefore electronically conducting. Top-down precursor 2H MoS_2_ is found naturally, while monolayer or few-layer 2H, 1T, and 1T’ polymorphs can only be synthesized artificially. Both the 1T and 1T’ phases are metastable, readily reverting back to the 2H phase given the thermodynamic conditions.

## 3 Characterization

**Figure 3:**
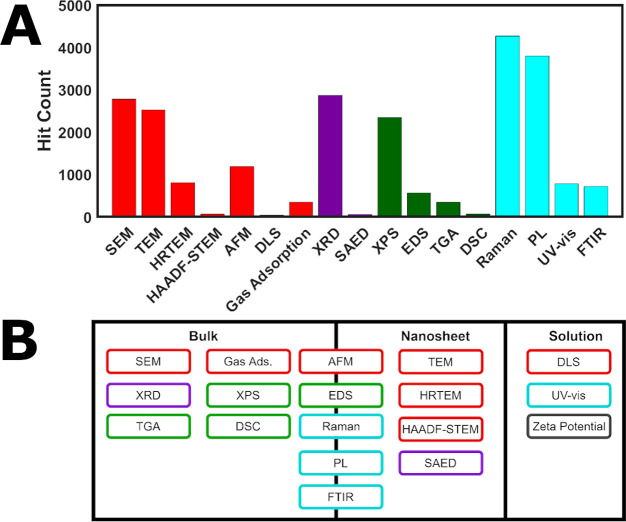
(A) Histogram of scan hits per relevant characterization category for 2011–2022 using Web of Science API (performed on 30/11/2022) and custom text classification. For detailed analysis methods, refer to the Supporting Information. Total count of 11,227 research articles presented, which can contribute to each of the characterization sub-categories. General color grouping of the techniques is based on the key information each technique provides regarding the material. (B) Schematic of different sample forms to which various characterization techniques can be applied. Abbreviations: Scanning electron microscopy (SEM), transmission electron microscopy (TEM), high-resolution TEM (HRTEM), high angle annular dark field STEM (HAADF-STEM), atomic force microscopy (AFM), gas adsorption (Gas Ads.), X-ray diffraction (XRD), selected area electron diffraction (SAED), X-ray photoelectron spectroscopy (XPS), energy dispersive spectroscopy (EDS), thermogravimetric analysis (TGA), differential scanning calorimetry (DSC), photoluminescence (PL), ultraviolet–visible spectroscopy (UV–vis), and Fourier transform infrared (FTIR).

Due to the wide range of production routes, varied conditions within manufacture routes, the polymorphism of MoS_2_, and the heterogeneity of the morphology that can be exhibited by MoS_2_ and MoS_2_ carbonaceous composites, a review of material characterization techniques is of extreme importance. Characterization techniques indicate the quality of production, MoS_2_ phase and its purity, morphology, degree of crystallinity, chemical environment, and can be used to explore reaction mechanisms. The material characterization techniques used within the surveyed literature of MoS_2_ or MoS_2_ carbonaceous composites have been categorized into five groups as shown in (Figure 3A). The broad categories focus on techniques that characterize the morphology, crystal structure, composition, and chemistry. Although certain techniques can provide relevant information toward multiple classification groups, they have been generally classified by the key information that the respective technique provides regarding the material. The dashed arrows in Scheme 1 indicate the various stages in the manufacturing process, where different characterization techniques are typically applied, to gain useful insight into the material properties and behavior. The benefits and limitations of each technique will be discussed in detail.

Finally, it is important to clarify some of the nomenclature regarding MoS_2_ sample characterization. In the literature, the top-down precursor MoS_2_ material is often referred to as bulk MoS_2_. However, this can lead to confusion because certain characterization techniques covered in this review can only be performed on bulk material forms. This by no means limits the analysis solely to top-down precursor MoS_2_, but rather signifies that the technique can only sample a relatively large material volume, such as a powder material made up of several milligrams. Thus, the use of the term bulk MoS_2_ will be avoided and replaced with top-down precursor MoS_2_. On the other hand, there are nanoscopic techniques which can be applied only to isolated nanosheets or stacks of nanosheets. A few techniques can also be performed on both bulk materials and isolated nanosheets. Lastly, there exist techniques that must be performed on solutions or dispersions. Figure 3B indicates the necessary sample form for each of the characterization techniques surveyed.

### 3.1 Morphology

**Figure 4:**
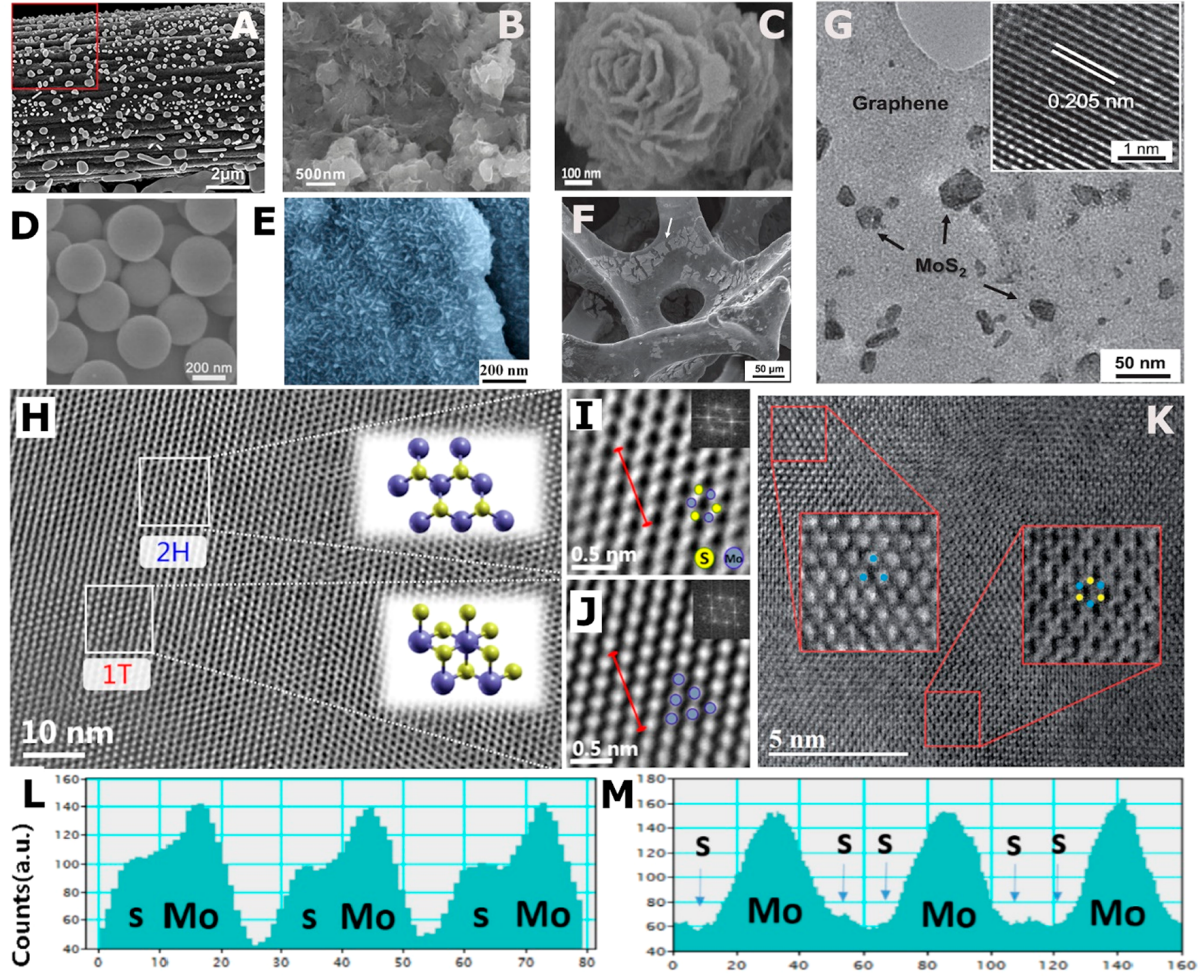
Microscopy techniques. SEM: (A) MoO_3_ on carbon filter paper. Adapted from ref (12). Copyright 2014 American Chemical Society. (B) MoS_2_/graphene composite (1:1). Adapted from ref (146). Copyright 2011 American Chemical Society. (C) MoS_2_/carbon nanoflowers. Adapted from ref (54). Copyright 2014 American Chemical Society. (D) MoS_2_ spheres. Adapted with permission from ref (38). Copyright 2018 Elsevier. (E) Perpendicular 1T MoS_2_ nanosheets on 2H MoS_2_ substrate. Adapted from (18). Copyright 2016 American Chemical Society. (F) MoS_2_ on a 3D graphene network. Adapted with permission from (74). Copyright 2013 Wiley. TEM: (G) MoS_2_ on a 3D graphene network. Adapted with permission from ref (74). Copyright 2013 Wiley. HRTEM: (H) heterogeneous structure of 1T/2H MoS_2_ nanosheet. Insets: structures of 2H and 1T MoS_2_. (I, J) Enlarged segment from H indicating the atomic arrangement of 2H and 1T MoS_2_ respectively. Insets: FFT of image. Adapted from ref (26). Copyright 2016 American Chemical Society. HAADF-STEM: (K) heterogeneous MoS_2_ produced from ball-milling. Adapted with permission from (110). Copyright 2016 Royal Society of Chemistry. For (H–K), blue and yellow atoms represent Mo and S, respectively. (L, M) Intensity distributions along lines in (I) and (J), respectively. Adapted from ref (26). Copyright 2016 American Chemical Society.

The multitude of possible MoS_2_ morphologies is one of the focal points of research interest, due to their influence on structural stability, reaction distance, and electrical conductivity for energy applications. Top-down synthesis methods generally deliver MoS_2_ nanosheets of varying quality,^6−9,26,102^ whereas bottom-up production methods typically offer the greatest morphology control. Purely liquid exfoliation, hydrothermal, and solvothermal production routes offer the greatest variety of morphologies (Figure 4A-G), including: 2H nanosheets,^6,35^ 1T nanosheets,^14,18,21,28,102^ doped nanosheets,^13,19,22,27^ nanoparticles,^12^ nanoflowers,^41,89^ nanorods,^35,108^ nanospheres,^38,58^ nanotubes,^64^ porous MoS_2_ structures,^64,123^ MoS_2_/graphene,^25^^,^^42^^,^^43^^,^^55^^,^^56^^,^^59^^,^^74^^,^^76^^,^^81^^,^^82^^,^^92^^,^^94^^,^^96^^,^^100^ MoS_2_/CNT,^30^^,^^31^^,^^45−51^^,^^60^^,^^61^^,^^93^ MoS_2_/polyaniline (PANI),^53,54^ MoS_2_/carbon,^36^^,^^40^^,^^44^^,^^57^^,^^78−80^^,^^84^^,^^85^^,^^87^^,^^90^^,^^91^^,^^99^ MoS_2_ on noncarbon-based supports,^15−17^ 1T MoS_2_/graphene,^10,37,83,103^ and 1T MoS_2_/carbon.^12,34,39,52,62,95,98^ The integration of MoS_2_ and a wide range of supports in electrodes not only increases the stability of MoS_2_ structures over long operating periods in harsh chemical environments,^6−64^^,^^72−103^ but also offers a highly conductive network. This leads to smaller resistance losses in electrochemical devices relative to the insulating top-down precursor MoS_2_.^106^

#### 3.1.1 Scanning Electron Microscopy (SEM)

SEM is one of the most widely applied morphology techniques within the surveyed literature (24.6% in Figure 3A) because it is relatively inexpensive, readily accessible, and works well with MoS_2_ samples as they are either conducting or semi-conducting. SEM functions by directing an electron beam onto the sample and detecting scattered electrons. These can be secondary or backscattered electrons. Secondary electrons occur when an incident electron knocks an electron out from the outter shells of an atom. Secondary electrons only occur near the surface of the sample, limiting the technique to the sample surface. When studying production routes, SEM can be implemented to identify intermediate products,^6,7^^,^^9−11^^,^^13−19^^,^^21−43^^,^^45−65^^,^^72−74^^,^^76−96^^,^^99−103^ for instance hydrothermally deposited MoO_3_ particles on carbon fiber paper (Figure 4A). More commonly SEM is applied to observe the morphology of the final synthesis products, such as MoS_2_/graphene nanosheets,^55^ forming MoS_2_/carbon nanoflowers,^54^ creating MoS_2_ spheres,^38^ attaching 1T MoS_2_ vertical nanosheets,^18^ or depositing MoS_2_ on a 3D graphene network^74^ (Figure 4B–F).

Additionally, SEM is often implemented to observe degradation effects in electrodes, as samples can easily be tested before and after cycling in batteries or electrolyzers,^68^ enabling the observation of material cracking or the loss of the typical top-down precursor MoS_2_ flake structure due to degradation. Unfortunately, air-free transfer into the SEM is challenging, and since some materials are air-sensitive, it is difficult to distinguish between surface changes due to air-free production and cycling or air exposure. Moreover, SEM is limited by the fact that it cannot resolve laterally small few-layered nanosheets and hence can only be used for the general surface morphology of bulk samples. Finally, all too often the SEM images displayed in publications are the single most favorable image taken, without providing a statistical analysis of the rest of the sample. The inclusion of a more detailed SEM approach within the article Supporting Information is encouraged. Ideally, this should include an image of the overall sample (*e.g.*, electrode segment), with the locations of interested highlighted and multiple magnified images of the areas of interest presented for statisitical significance.

#### 3.1.2 Transmission Electron Microscopy (TEM)

TEM is another widely used microscopy technique within the surveyed literature (23.3% in Figure 3A), whereby an incident electron beam transmits through an extremely thin sample and is detected on the other side. TEM allows for single nanosheets or stacks of several nanosheets to be observed (Figure 4G), and therefore can confirm the quality of the nanoscopic MoS_2_ products.^6,7^^,^^9−11^^,^^13−19^^,^^21−43^^,^^45−65^^,^^72−74^^,^^76−96^^,^^99−103^ Studies not focusing on reporting the development of material synthesis, utilizing only top-down precursor MoS_2_ do not require TEM, unless it is used to contrast a key change in the material, *e.g.*, observing the degradation effects of electrochemical testing during *in situ* studies.^42,125,143^ Nevertheless, TEM suffers from major setbacks such as extensive operating time, additional processing steps including material dispersion and grid preparation, inability to directly image thicker structures such as electrodes (Scheme 1), and most importantly only sampling a minute fraction of the entire bulk sample. Therefore, it is strongly recommended to report the count of observations and length measurements undertaken for TEM statisitics and follow a similar sampling protocol as was suggested for SEM Supporting Information. Additionally, the TEM electron beam can interact with the material leading to degradation of the sample and structural changes, such as phase shift within the nanosheets.^125^

#### 3.1.3 High-Resolution TEM (HRTEM) and High-Angle Annular Dark-Field Scanning TEM (HAADF-STEM)

HRTEM and HAADF-STEM are techniques that provide a further step up in magnification, allowing the observation of the atomic structure of MoS_2_ (HRTEM Figures 4H–J and HAADF-STEM Figure 4K). 2H MoS_2_ has a hexagonal arrangement (Figure 2B), which can be identified in microscopy by a repeating hexagonal structure with a central gap (Figure 4I and K). On the other hand, 1T MoS_2_ is more difficult to observe due to the misalignment of the S and S’ atoms (Figure 2B). This results in an apparent closer packed structure, observed via microscopy as the filling of the central gap (Figure 4J). Typically, both 2H and 1T MoS_2_ are present in a heterogeneous structure during microscopy,^26,110,125^ as seen in both Figure 4H and K. To further justify the phase identification with HRTEM or HAADF-STEM, intensity distributions can be measured along the lines shown in Figure 4I and J. If the resulting repeating pattern exhibits a shoulder (Figure 4L) the phase is 2H,^25,26^ as the shoulder indicates the protruding S atoms being detected in addition to Mo. However, if the repeating peak pattern is composed of a single peak (Figure 4M), only the Mo atoms are detected, indicating the 1T phase.^125^

#### 3.1.4 Atomic Force Microscopy (AFM)

**Figure 5:**
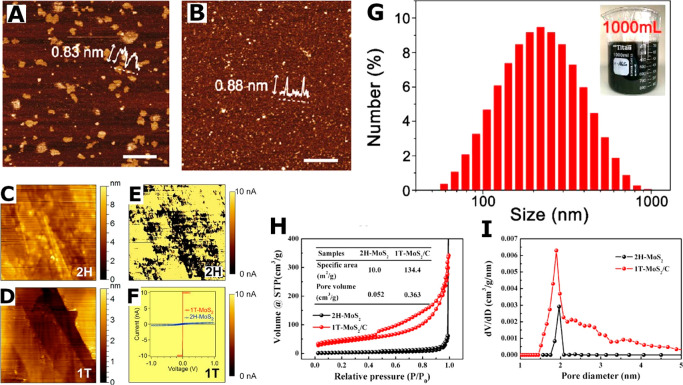
Morphology techniques. AFM: (A) and (B) of quantum dots exfoliated from Li electrochemically intercalated in MoS_2_ with current densities of 1.0 A g^–1^ and 0.001 A g^–1^, respectively. Scale bars are 200 nm. Adapted from ref (9). Copyright 2018 American Chemical Society. CS-AFM: (C, D) micrographs of 2H MoS_2_ from CVD and 1T MoS_2_ from lithiation and exfoliation of the 2H MoS_2_. (E, F) Conductivity maps of (C) and (D), respectively. Inset in (F): current–voltage sweeps of 2H and 1T MoS_2_. Adapted with permission from ref (24). Copyright 2013 American Chemical Society. DLS: (G) size distribution of liquid-exfoliated MoS_2_. Inset: exfoliated MoS_2_ solution. Adapted with permission from (29). Copyright 2018 Elsevier. N_2_ adsorption: (H) adsorption/desorption isotherms and (I) pore diameter distribution for 2H MoS_2_ and 1T MoS_2_/carbon composite. Inset in (H): table of BET and BJH values. Adapted with permission from ref (39). Copyright 2019 Wiley.

AFM is another heavily used morphology technique from the surveyed literature (10.5% in Figure 3A), which enables the measurement of lateral dimensions and sample thickness. AFM relies on a cantilever with an atomically sharp tip. The tip is scanned across the surface of the sample resulting in repulsive forces between the sample and tip. Deflections in the cantilever are measured by reflecting a laser off the top of the cantilever onto a position-sensitive photodetector. The photodetector measures a voltage generated by the reflected laser. Subsequently, a proportional integral derivative feedback loop is used to control the height of the cantilever via a piezoelectric material. Thus, AFM can map the surface of a sample of uniform thickness, and when nanosheet or few-layer MoS_2_ is deposited on a suitable substrate (typically cleaved atomically flat mica or graphite), its width and thickness can be measured (Figure 5A and B). Alternatively, AFM can be used to map the morphology of electrodes, in both pristine and cycled forms (Scheme 1).

AFM is generally limited by slow imaging time and a relatively small scanning area, on the order of magnitude of 10 μm^2^, whereas other techniques such as SEM can cover several mm^2^ in a single image with a quick scan time. Like TEM, HRTEM, and HAADF-STEM, AFM also struggles with the fact that the measurements made, even of 100 nanosheets, may not be representative of the entire sample. Subsequently, AFM has a small statistical representation of the sample analyzed, often involving tedious and time-consuming manual measurements of nanosheet dimensions one at a time. As a result, out of the 16 manually surveyed papers, not a single study reported the number of MoS_2_ nanosheets/flakes measured. In other studies, the reported measurement count was between 50 and 100 nanosheets or flakes.^109,116^ Therefore, we strongly recommend including the measurement count in future articles to satisfy statistical significance.

The thickness of a single MoS_2_ nanosheet is a disputed topic with papers surveyed reporting values in the range 0.7–1.2 nm.^6^^,^^8−10^^,^^15^^,^^20^^,^^21^^,^^24−26^^,^^28^^,^^29^^,^^57^^,^^77^^,^^87^^,^^88^^,^^116−118^^,^^126^ Although overall sample thickness distributions between production routes may vary, for single nanosheet measurements, Eda et al.^116^ claim that the discrepancy between mechanical exfoliation reports (∼0.7 nm) and their *n*-butyl-lithium fabricated nanosheets (1.0 nm − 1.2 nm) arises from surface corrugation through distortions, absorbents or entrapped solvent molecules in the final material. The data gathered in this review was insufficient to propose a standpoint on whether single nanosheet measurements vary due to the differences between production methods, or experimental setup discrepancies such as the use of a substrate in AFM or piezoelectric material calibration. Therefore, we believe an in-depth study across a multitude of production routes, substrates, and AFM instruments is required to validate the current hypotheses.

The lateral dimensions of single exfoliated nanosheets vary significantly compared to the thickness, with the smallest sheets bearing a lateral size of 14.6 nm and the largest nanosheets spreading 3 μm.^9,25^ However, due to the lateral resolution of AFM being limited by tip-convolution, values near the lower bound are subject to larger experimental error. Unlike thickness, the lateral dimensions are confirmed to be heavily dependent on the production method used. Some methods preserve large flake sizes as they are relatively gentle on the materials (*e.g.*, solid state), whereas other methods fragment the flakes into very small nanosheets (*e.g.*, ball milling coupled with *n*-butyl-lithium). Using different current densities in a lithium electrochemical cell, the lateral dimensions of MoS_2_ quantum dots were varied between Figure 5A and B, yet the thickness remained in the same range.^9^ Depending on the distinct requirements of energy applications, different sized nanosheets are preferred. Generally, smaller nanosheets (both in lateral and thickness dimensions) allow for faster reaction kinetics due to shorter diffusion paths, and enhanced surface area. Thus, smaller nanosheets are sought out for HER catalysis and power batteries. However, recent computational modeling and statistical analysis suggest that 2D materials are not desirable in batteries due to hindered rate performance.^145^

Current sensing AFM (CS-AFM) (Figure 5C–F) allows for the simultaneous measurement of the surface morphology using conventional AFM and the change in current between the AFM tip and the sample material under constant voltage. In addition to the thickness (Figure 5C and D), this enables 2H and 1T MoS_2_ to be easily distinguished in heterogeneous samples, since 2H is semi-conducting (Figure 5E), while the 1T metallic phase is conducting^24^ (Figure 5F). The inset in Figure 5F highlights the distinction in current exhibited between the two phases as a voltage range is explored.

In contrast to conventional AFM, high-speed AFM (HS-AFM) relies on the use of a vibrometer to measure sample height using light reflected off the cantilever, avoiding the use of a feedback loop coupled with a piezo-electric material. This eliminates the need for periodic height calibration of the piezo-electric material and significantly improves the scan rate and image size. Hence, multiple images can be stitched together to cover a sample area of approximately 10 μm^2^ with HS-AFM. One study measured 824 nanosheets using HS-AFM,^121^ increasing the nanosheet count 8-fold relative to standard AFM studies.^111,118^ Although this technique has not been applied to MoS_2_, it has been used to study mechanically cleaved graphite and metal-ammonia method synthesized graphene, TiS_2_, MoSe_2_, and Bi_2_Te_3_ nanosheets.^121^ The total number of HS-AFM height measurements performed on the samples varied from 1500 to 14,400, offering the potential for faster augmentation of statistically significant AFM thickness measurements.

#### 3.1.5 Dynamic Light Scattering (DLS)

DLS is a rapid solution-based characterization technique which allows for measurement of the lateral size distribution (Figure 5G) of a dispersion. Due to its short operating time span of several minutes, DLS can be repeated many times to represent a large proportion of the original sample, and the number of scans should be reported clearly in scientific methods. Microscopy techniques such as TEM and AFM rely on user input to determine a diameter equivalent to the lateral dimensions of nanosheets; in contrast, DLS is programmed to directly measure the hydrodynamic diameter of particles. A hydrodynamic diameter corresponds to a theoretical sphere of the same translational diffusion coefficient as the nanosheet or particle measured, and will therefore differ from observations made with microscopy techniques. DLS can only detect a particle size range of 10–200 nm.^147^ With regards to nanosheet thickness, the combination of reduced detection range and the influence of the hydrodynamic diameter make it impossible for DLS to be used to capture the thickness of exfoliated nanosheet MoS_2_.

#### 3.1.6 Gas Adsorption

Gas adsorption methods can measure the surface area and pore size distribution of a powder material (Scheme 1 and Figure 3B). Nitrogen gas is commonly used for its inert nature and therefore will not react with the sample. Instead, N_2_ will only adsorb onto the surface. Plotting the adsorption isotherm as seen in Figure 5H, the Brunauer–Emmett–Teller (BET) specific surface area (m^2^ g^–1^) and pore volume (cm^3^ g^–1^) can be calculated, allowing for improvements in available surface area and porosity via processing or bottom-up manufacture to be confirmed.^11^^,^^14^^,^^17^^,^^18^^,^^27^^,^^32^^,^^34^^,^^35^^,^^37−40^^,^^44^^,^^47^^,^^49^^,^^50^^,^^54^^,^^58−60^^,^^63^^,^^73^^,^^78^^,^^82^^,^^85^^,^^88^^,^^94^^,^^97−99^ Barret–Joyner–Halenda (BJH) analysis (Figure 5I), allows for the pore size or pore size distribution (nm) in a material to be identified.^11^^,^^17^^,^^18^^,^^32^^,^^34^^,^^35^^,^^37^^,^^38^^,^^40^^,^^47^^,^^49^^,^^50^^,^^78^^,^^82^^,^^85^^,^^88^^,^^94^^,^^99^ The surface area of 2D materials is claimed to be one of the key material properties beneficial for energy applications. However, often pore size distributions are rarely presented, despite being crucial experimental data for application modeling.^148,149^

### 3.2 Crystal Structure

#### 3.2.1 X-ray Diffraction (XRD)

**Figure 6:**
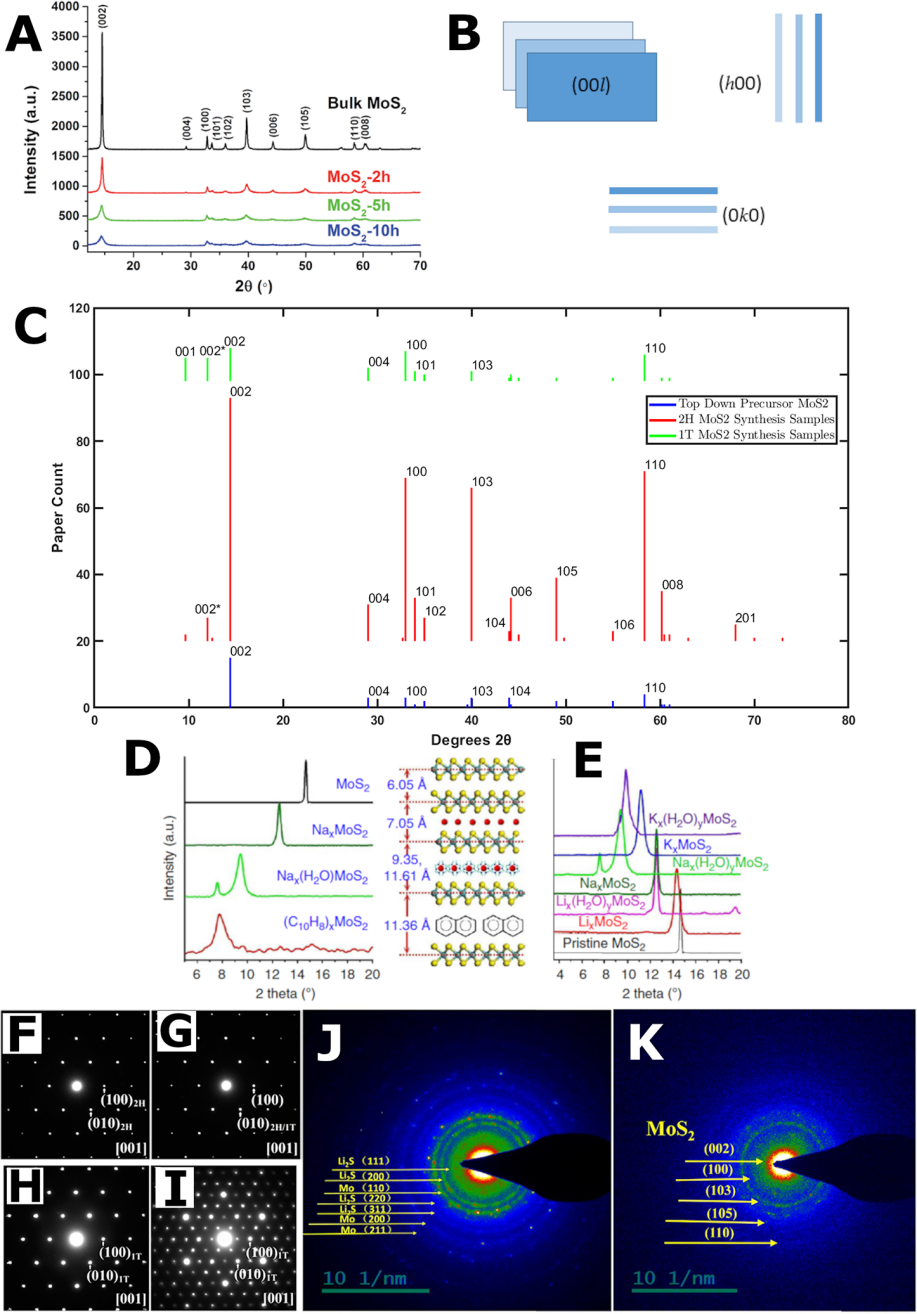
Crystal structure techniques. XRD: (A) Diffraction patterns of top-down precursor MoS_2_ ball-milled for 2, 5, and 10 h. Adapted with permission from ref (110). Copyright 2016 Royal Society of Chemistry. (B) Schematic representation of a single flake of MoS_2_ viewed from the direction of the different Miller indices plane groups. (C) Statistical analysis of the MoS_2_ XRD peaks from literature. Note, the 2θ positions are not exact. Approximations and averages were used where literature was vague. Additionally, the intensity is not representative of an XRD pattern but the number of literature sources reporting the different peaks. Only peaks with more than five mentions were indexed. XRD: (D, E) diffraction patterns of intercalation of species in the MoS_2_ lattice using sodium naphthalenide. Schematic displays the varying degree of interlayer space expansion due to different intercalants. Adapted with permission from ref (118). Copyright 2014 Nature. SAED: patterns of commercial MoS_2_ powder in Na cells discharged to (F) 60, (G) 80, (H) 160, and (I) 256 mAh g^–1^. Adapted from ref (75). Copyright 2014 American Chemical Society. (J, K) SAED: of 1T MoS_2_/graphene electrodes in a LIB after the (J) third lithiation (discharge) and after the (K) third delithiation (charge), respectively. Adapted with permission from ref (37). Copyright 2019 Elsevier.

In XRD, monochromatic X-rays are diffracted by the crystal lattice of a powder sample. Powder samples, and electrodes formed from powders, are usually assumed to be composed of significant amounts of particles arranged in every possible orientation with respect to the incident beam. Thus, for the range measured, the resulting diffraction pattern contains reflections associated with each *d*-spacing in the crystal lattice. XRD is a bulk technique (Figure 3B), which identifies different crystalline materials contributing to the overall composite powder, and the substrate in electrodes (Scheme 1). XRD can be used to distinguish between crystalline materials which give rise to sharp peaks (Figure 6A), or amorphous materials that give rise to less intense broad peaks. XRD is one of the oldest characterization techniques applied to MoS_2_ samples. In 1959, XRD was used by Rüdorff to confirm intercalating MoS_2_ with Li metal in liquid ammonia to form Li_*x*_MoS_2_ salt,^119,150^ and in 1992 XRD was used to identify 1T MoS_2_.^127^

Due to its wide availability, XRD is another commonly applied characterization technique within the surveyed literature (25.5% in Figure 3A). Therefore, there is abundant literature to compare against both in XRD databases, as well as current and older journal articles. In publications it is extremely beneficial to include top-down precursor MoS_2_ XRD patterns as a reference for all synthesis methods, as well as diffraction patterns of the reactants utilized in bottom-up synthesis methods. XRD is often carried out on a powder sample in air, on an air free powder covered by Kapton tape^59,129,151^ or Parafilm,^66^ inside a sealed glass capillary,^152^ or *in situ* within an electrochemical device.^67,143^ Clear and concise experimental details and substantial data facilitates the comparison of future samples with the literature and clarifies any differences observed in manufacturing.

Figure 6A presents the XRD pattern of a typical crystalline top-down precursor MoS_2_ measured with a Cu Kα X-ray source (λ ∼ 1.54 Å). Out of the 91 papers manually surveyed presenting XRD data, 79% reported the XRD source used and predominantly employ a Cu Kα X-ray source (76%), as reported previously.^152^ However, 21% of studies did not report their X-ray source, and therefore we strongly encourage for more detailed reporting of experimental conditions. The top-down precursor MoS_2_ diffraction pattern in Figure 6A exhibits many sharp peaks identifying the crystalline nature of the sample. Notable MoS_2_ peaks are indexed according to their respective d_hkl_-spacing, which indicate the distances between the different planes of atoms in the crystal. Figure 6B schematically represents the nanosheets within a single flake of MoS_2_ as observed from the viewpoint of different *d*-spacings. The major peak in top-down precursor MoS_2_ corresponds to the 002 reflection (Figure 6A), signifying the interlayer space between the layers of a 2H MoS_2_ flake, and all 00*l* plane reflections are higher order reflections of the 002 plane.

Generally, in reflex geometry XRD the 00*l* family indicates the preferential orientation of layered materials due to their flake-like or nanosheet morphology. For top-down precursor MoS_2_ powders, the prevailing detection of 00*l* planes indicates that the MoS_2_ flakes are positioned flat on top of the substrate^153,154^ (Figure 6B). This is particularly the case when nanosheet MoS_2_ is deposited from a dispersion, as the lateral dimensions of the nanosheets relative to thickness is significantly larger and therefore it is difficult for nanosheets or flakes to stabilize perpendicular to the substrate.^153^ Both 100 and 010 reflections depict intralayer spacing within a single nanosheet. Their respective family groups *h*00 and 0*k*0 represent a MoS_2_ flake sitting vertically on the substrate so that the nanosheet interlayer spaces are directly exposed (Figure 6B). Reflections that are a combination of 00*l* and another direction, such as the *h*0*l* or 0*kl* families, represent MoS_2_ flakes sitting flat on the substrate but are slightly offset and therefore are not perfectly parallel to the substrate. Their strong presence is expected in powder samples, as all orientations are assumed to be present in bulk powders.

Figure 6C displays statistical analysis of the manually surveyed literature XRD peaks reported for various phases and forms of MoS_2_ samples. Each count represents a paper that has reported the peak for 2H top-down precursor MoS_2_, 2H MoS_2_ structures, or 1T MoS_2_ structures and composites. Therefore, by contrasting the intensities Figure 6C can be used to suggest any dominant orientations^153,154^ or trends in the literature reporting of different sample forms. From the statistical analysis, all three sample forms indicate a preferential flat stacking orientation^153,154^ due to a strong 00*l* family. Additionally, all sample forms also exhibit *h*00, *h*0*l*, and *hk*0 orientations, with 2H MoS_2_ experiencing the largest reporting. If a single MoS_2_ flake can be represented as a deck of cards, then exfoliated samples can be described as a set of cards which has fallen on the floor. Cards will stack and overlap vertically, while displaying a disorder of directions laterally.^153,154^ Similarly, if nanosheets are deposited, they will preferentially position flat on the surface and stack vertically on top of other nanosheets, yet they will be laterally largely misaligned.^153^ Furthermore, for 2H MoS_2_ the presence of sharp 010 and 100 reflections signifies in-plane sample crystallinity.

Processing top-down precursor MoS_2_ during exfoliation routes may have different effects on the observed XRD peaks. For instance, mechanically exfoliating MoS_2_ via ball milling^110^ shown in Figure 6A results in no noticeable shift in peak positions, yet the intensities of the peaks decrease significantly as the ball milling time is increased. Additionally, the originally sharp peaks broaden with time. The Scherrer equation (eq 1), can be used to explain this difference, where τ is the mean size of the ordered crystalline domain, κ is the shape factor, λ is the incident XRD wavelength used, β is the broadening of the peak at full-width half the maximum (fwhm), and θ is the Bragg angle.^153^ The Scherrer equation dictates that the diffraction peaks of weaker intensities and larger fwhm have smaller crystal domains.^153,154^ Therefore, as the lateral size of MoS_2_ flakes reduces during ball-milling, the respective XRD peaks decrease in height while increasing in width.Scherrer equation:
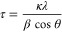
1Rüdorff demonstrated that intercalating various alkali metal cations into the interlayer space of top-down precursor MoS_2_ results in the 002 peak shifting to a lower angle due to the expansion of the interlayer spacing (Figure 6D and E).^118,119,121^ The larger the intercalating cation the bigger the down-shift of the 002 peak. It is also possible for the solvent to be co-intercalated inside the gallery space alongside the intercalating cations or trapped during drying and restacking resulting in an even bigger spacing^118^ (Figure 6D and E). This phenomenon is referred to as the entrapment effect. Hence, within the metal-ammonia solution intercalation method, Li surprisingly leads to the largest increase in the interlayer spacing despite exhibiting the smallest cation radius because it co-intercalates ammonia^119^ (NH_3_). The phenomenon of intercalation is not restricted to alkali metals alone; transition metal cations have also been intercalated,^7^ and organic solvents have formed sandwich-like composites due to stacking^118^ resulting in even larger lattice *d*-spacings (Figure 6D).

Out of all the top-down precursor MoS_2_ peaks (Figure 6A), the one with the highest intensity is the 002 reflection located at median 14.4° (standard deviation ±0.88°) when measured with a Cu Kα X-ray source (λ ∼ 1.54 Å).^8−15^^,^^17−20^^,^^24^^,^^25^^,^^27−29^^,^^31^^,^^32^^,^^34−41^^,^^43−45^^,^^47^^,^^48^^,^^50^^,^^52−56^^,^^58^^,^^59^^,^^62−65^^,^^67^^,^^68^^,^^71^^,^^73^^,^^75−81^^,^^83^^,^^84^^,^^86^^,^^88−94^^,^^96^^,^^98^^,^^100^^,^^102^^,^^103^ This corresponds to a *d*-spacing of approximately 0.62 nm in the top-down precursor MoS_2_.^58,71,86^ In most of the 2H literature samples surveyed, little change in the 002 peak post manufacture relative to the top-down precursor MoS_2_ was observed, with a median 002 position at 2θ values of ∼ 14.2° (standard deviation ±0.22°) and median *d*-spacing of 0.62 nm.^28^^,^^35^^,^^39^^,^^51^^,^^54^^,^^55^^,^^59^^,^^61−63^^,^^79^^,^^81^^,^^89^ For interlayer-expanded final synthesis products containing 1T MoS_2_, the two most expanded interlayer samples surveyed exhibit 001 peaks shifted to 7.36° and 7.5°, respectively, when measured with a Cu Kα X-ray source.^34,102^

High magnification microscopy techniques (HRTEM and HAADF-STEM) allow for the measurement of lattice fringes, usually carried out at the nanosheet edge, which are equivalent to the distances between atoms within a single plane or between stacked nanosheets (inset Figure 4G). Our survey of 49 HRTEM measurements, from predominantly hydrothermal and solvothermal synthesis, found the (002) fringe reported with median 0.63 nm (standard deviation ±0.03 nm). Similarly, analyzing 20 reports of 2H MoS_2_ XRD measurements found the median 002 *d*-spacing to be 0.62 nm (standard deviation ±0.02 nm), in close agreement with the HRTEM statistics. For the statistical calculations, samples with a significant interplanar spacing (>0.7 nm),^35^^,^^37^^,^^39^^,^^52^^,^^58^^,^^60^^,^^62^^,^^64^^,^^84^^,^^89^^,^^94^^,^^98^ were not included. Thus, measurements of lattice fringes can be used in combination with XRD to provide two separate points of characterization for synthesis routes. Nevertheless, out of 86 2H MoS_2_ XRD samples surveyed,^6−8^^,^^10,11^^,^^14−16^^,^^18^^,^^20−29^^,^^32−50^^,^^52^^,^^53^^,^^55−58^^,^^60−65^^,^^72−74^^,^^76−80^^,^^82−91^^,^^94−96^^,^^98−102,105^ the respective authors claim that HRTEM findings agree for only 15 samples. However, fewer studies confirm results through direct quantitive comparison.^28^^,^^35^^,^^55^^,^^61^^,^^63^^,^^79^^,^^81^^,^^84^^,^^89^^,^^94^

Due to the possibility of interlayer spacing expansion, the nomenclature in the literature for the shifted 2H 002 peak is 002’. Although the 002’ XRD reflection is associated with a 2H ABABAB stacking structure (Figure 2A), and the 001 reflection represents the AAAAAA stacking order of 1T (Figure 2C), one cannot distinguish between the two XRD peaks. The peak position depends on the intercalant and co-intercalated solvent, or other means of interlayer space expansion. Additionally, in heterogeneous phase samples, both expanded peaks can coexist with the 2H 002 peak in experimental diffraction patterns. This occurs when only part of the MoS_2_ structure is intercalated or otherwise expanded. Moreover, all other top-down precursor MoS_2_ peaks can be found in both 2H and 1T exfoliated powders, preventing any further peak changes to allow clear phase identification. Hence, we believe that supporting proof from other experimental techniques is required for MoS_2_ phase confirmation.

#### 3.2.2 Selected Area Electron Diffraction (SAED)

SAED is carried out in a TEM or STEM setup. Similar to XRD, SAED enables the classification of crystalline materials. However, XRD covers a bulk area of the sample in the millimeter range, whereas SAED examines the nanometer scale of TEM operation. Therefore, SAED yields little statistically significant information on the overall sample but can provide high quality information regarding localized sample segments. On the other hand, XRD represents the sample average. A SAED detector is similar to that of an XRD, the only difference being that XRD literature presents the integration of the detector as a diffraction pattern, whereas SAED literature publishes a 2D image of the detector. For highly crystalline materials in a single orientation, the undiffracted beam is observed by the detector, surrounded by Laue spots in a regular array pattern (Figure 6F–I). If the sample contains multiple orientations of the same material, clear concentric rings are observed (Figure 6J and K). In both cases, the distance from the center of the detector to the Laue spots or rings corresponds to reflections (Figure 6I). In the case of amorphous samples with similar bond lengths, a diffuse halo may be observed.

Figure 6F–I shows the SAED patterns of a MoS_2_ SIB electrode at different states of discharge.^75^ They include 2H MoS_2_, a heterogeneous structure of 2H and 1T MoS_2_, 1T MoS_2_, and the distorted 1T’ structure with Mo zigzag chains.^75^ In this case, a HAADF-STEM setup was used for the SAED patterns, whereby the pure microscopy images revealed the distinct phases identified by the key signatures discussed in the morphology section 3.1.3. The SAED patterns for 2H, heterogeneous 1T/2H and 1T are identical, varying only in intensity but exhibiting the same reflection indexes.^75^ Only the distorted 1T’ displays a different Laue spot arrangement (Figure 6I). Between the high magnification microscopy images and the SAED patterns, the local phase can be confirmed. Since SAED is an accompanying technique to high magnification microscopies, its key drawback is that it represents only a small statistical segment of the overall sample.

#### 3.2.3 Fast Fourier Transform (FFT) Analysis of TEM Micrographs

Another useful technique for analyzing crystallinity in a localized manner like SAED, is the fast Fourier transform (FFT) analysis of TEM micrographs. The distinction between the two techniques is that SAED is a direct measurement of the local structure diffraction pattern, whereas FFT is a computational analysis carried out on a TEM or STEM image. Thus, applying FFT in real-time while viewing TEM samples enables a researcher to identify the material observed, or retrospectively analyze exported TEM images. The insets in Figure 4I and J represents the FFT of their respective HRTEM images. The output of FFT is similar to that of SAED, whereby concentric rings or dot patterns correspond to reflections of crystalline materials. Generally, both SAED and FFT are used frequently as supporting secondary sources to validate bulk XRD findings, as they suffer the same disadvantage as TEM with small sample representation and low statistical significance. Therefore, we suggest crystallographic characterization techniques be used in tandem to support findings, and that the breadth of the sample scanned by any one technique be closely considered.

### 3.3 Composition

#### 3.3.1 X-ray Photoelectron Spectroscopy (XPS)

Due to the various manufacturing routes and composites made using MoS_2_, establishing the composition and phase of the material is key to understanding its behavior within energy applications. XPS is the most widely used compositional analysis technique from the surveyed literature (20.5% in Figure 3A), applying to both powder and electrode sample types (Scheme 1). In XPS, incident X-rays interact with electrons in the core–shells of atoms. Electrons are expelled from their orbitals due to the X-ray photon interaction. The holes created by expelled electrons are filled by electrons relaxing from higher energy orbitals, which emit X-ray photons in the process to conserve energy. The newly emitted photons can then interact with an outer electron, causing secondary emissions. These lower energy emitted electrons are known as Auger electrons. The kinetic energy and electron count of both primary emitted electrons and Auger electrons are measured in XPS. Elements and their oxidation states can then be deduced from peak energy positions of the detected electrons, as the electron core orbital energies are tabulated in large databases. However, deducing elemental information regarding compounds and composites becomes difficult due to overlapping peaks, the possibility of contaminants, and the formation of unexpected by-products in local amounts.

**Figure 7:**
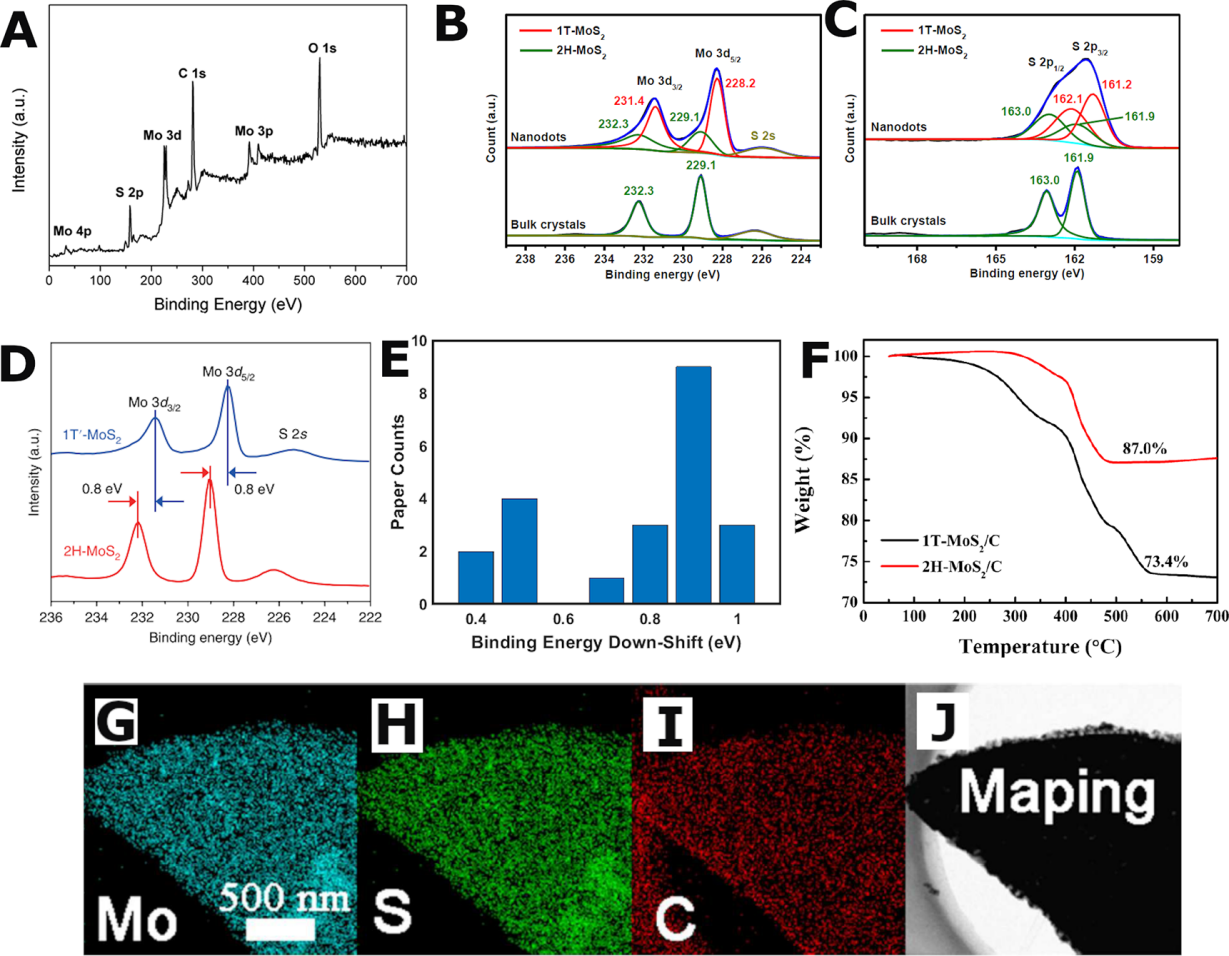
Composition techniques. XPS: (A) survey scan of MoS_2_ grown vertically on graphene sheets. Adapted from ref (56). Copyright 2016 American Chemical Society. (B, C) Fitting of the Mo 3d and S 2p peaks with models to quantify the transition from 2H top-down precursor crystals to 1T nanodots. Adapted with permission from ref (25). Copyright 2018 Wiley. (D) Mo 3d region scans of 2H and 1T’ MoS_2_ crystals. Adapted with permission from ref (20). Copyright 2018 Nature. XPS statistics: (E) bar chart of the reported energy down-shift of the Mo 3d peaks for 2H to 1T phase shift in literature. TGA: (F) 2H and 1T MoS_2_/C composites. Adapted with permission from ref (39). Copyright 2019 Wiley. EDS: mapping of MoS_2_/carbon corn stalk composites for elements; (G) Mo, (H) S, and (I) C. (J) The original SEM image. Adapted from ref (36). Copyright 2018 American Chemical Society.

If interpreted correctly, XPS can be beneficial in identifying the composition and structure of the material. Figure 7A shows a survey spectrum, a scan covering a wide binding energy range allowing for identification of the elements within the sample by careful peak fitting. The sample observed is a composite of MoS_2_ and graphene, in which sheets of MoS_2_ are vertically anchored on larger graphene sheets.^56^ As seen from the spectrum, there exist several ranges of interest where peaks are observed. First, between 155 and 170 eV the S 2p region is found with doublet peaks representing S^2–^. Second, the Mo 3d region between 220 and 240 eV is observed, where the Mo doublet peaks are located. This region is the most often analyzed region in MoS_2_ energy application articles. Third, the carbon C 1s region found in the range 280 eV – 300 eV is of paramount importance as it is the peak against which the binding energy scale of the entire spectrum is calibrated. This data analysis procedure is standardly used as most XPS samples will have a layer of adventitious carbon on their surface. This region is also very important to MoS_2_ composites as much of the literature focuses on the combination of MoS_2_ and carbon to obtain an active material deposited on a more conductive carbon substrate or uses carbon as a conductive additive. However, contributions to the C 1s region such as C–O–Mo^39,62^ are difficult to distinguish from the complex adventitious carbon spectrum, as the adventitious fingerprint varies from sample to sample. Due to the nature of some MoS_2_ work being air sensitive or originating from molybdenum oxide precursors, it is always worth investigating the O 1s region located in the range 524 eV – 544 eV. Finally, a further area of interest can also be the Li 1s region in the range 50–62 eV, as many production routes or applications depend on MoS_2_ and Li interactions. However, since Li is a very light element the sensitivity of XPS to Li detection is rather low.

The survey spectrum (Figure 7A) allows for the majority of the elements in the sample to be identified or postulated and thus a general composition of the material is formed by fitting each of the peaks with a peak model. To assist in such an endeavor, median 2H and 1T values and other useful literature peaks are reported in Table SI 6. To gather further information about the material, such as the environment of the elements in the sample, it is necessary to consider the individual scans of the elements of interest and not only the survey spectrum. Peaks will change their position based on the chemical environment, state, and phase. As already mentioned, in the case of MoS_2_ these correspond to the Mo 3d and S 2p regions (Figure 7B–D). MoS_2_ structures are characterized by the Mo^6+^ 3d_3/2_, Mo^4+^ 3d_3/2_, Mo^4+^ 3d_5/2_, and S^2–^ 2s peaks in the Mo 3d region (Figure 7B and D) and the S^2–^ 2p_3/2_ and S^2–^ 2p_1/2_ peaks in the S 2p region (Figure 7C), which are summarized in Table SI 6. Note that the Mo^6+^ 3d_3/2_ corresponds to the Mo–O bond.^38,56,101^ Often, the intensity of the Mo^6+^ 3d_3/2_ peak is relatively low compared to the other Mo peaks, though it increases with oxygen from air exposure, cycling, or O_2_ bombardment.^101,124,126^ Furthermore, a wide range of other commonly observed XPS peaks are reported in Table SI 6 to help distinguish the chemical bonding within composite materials.

When calculating the local composition of a heterogeneous sample, careful peak fitting of the previously described XPS regions must be performed. In addition, XPS often aids in distinguishing the 2H and 1T phases of MoS_2_. The transition from the semi-conducting 2H to the metallic 1T is accompanied by a down-shift in the binding energy of the electrons in the Mo^4+^ 3d_5/2_ and 3d_3/2_, and S^2–^ 2p_3/2_ and 2p_1/2_ states. Figure 7D shows the difference between the two phases, whereby the metastable 1T’ crystal is annealed to revert to 2H. Typically, the down-shift in binding energy from 2H to 1T is approximately 0.9 eV^8^^,^^12^^,^^14^^,^^18^^,^^19^^,^^25^^,^^39^^,^^52^^,^^64^^,^^98^^,^^101^^,^^104^ (Figure 7E).

To the best of our knowledge, a pure 1T MoS_2_ sample has not been synthesized to date. To establish the relative amounts of 2H and 1T MoS_2_ within a single sample, a peak fitting of the Mo 3d and S 2p regions must be carried out (Figure 7B and C), by fitting a total of four peaks for each of the two phases.^20,25^ Purity ranges from 20% to 94% 1T phase,^7−10^^,^^12^^,^^14^^,^^21^^,^^25^^,^^37^^,^^98^^,^^110^^,^^122^^,^^126^ with no visible connection to production route category. Since peak fitting techniques are not universally standardized, the 1T phase purity analysis is subject to significant operator bias. Furthermore, XPS conducts point measurements within a significantly larger sample, and thus unless the sample is homogeneous extrapolation from a point scan to the entire sample is highly error-prone. Therefore, to establish higher statistical significance multiple elemental and survey scans need to be carried out, across multiple locations within the sample. Finally, although XPS is a sample surface technique it is possible to excavate into the sample using ion bombardment to etch away the surface. However, weak Ar^+^ bombardment results in a binding energy down-shift from 2H to 1T.^108,131^ It remains unclear whether having a powerful enough ion beam will simply uncover the intact and unaltered phase underneath or result in 2H converting to 1T during the etching process.

#### 3.3.2 Thermogravimetric Analysis (TGA)

TGA (or TG) uses the mass change of powder samples as a function of increasing temperature to determine sample composition. Mass changes for certain chemical species occur at combustion point temperatures (in air) or decomposition point temperatures (in noble gases), and therefore can be used to quantitatively determine the sample composition. In dynamic TGA, the temperature is increased linearly, and the mass of the sample is monitored (Figure 7F). As the temperature is raised in a pure MoS_2_ sample, MoS_2_ will be oxidized in the range 300–500 °C, whereby the sulfur will form a gaseous product and the molybdenum oxide (MoO_3_) will be the only solid remnant left behind. However, in most instances, TGA is used to establish the composition of MoS_2_ in carbon composites and is operated in the range 25–700 °C^20^^,^^36^^,^^37^^,^^39^^,^^40^^,^^43^^,^^48−50^^,^^58^^,^^61^^,^^76^^,^^78^^,^^99^^,^^100^^,^^122^ (Figure 7F). Carbon-based materials form CO_2_ during the measurement. It is therefore paramount to clearly state the underlying assumptions on which MoS_2_ TGA analysis is based. Often it is assumed that only pure MoO_3_ remains after the heat treatment, which may not be accurate in the presence of carbon. Therefore, we believe that TGA is a good supporting technique, which should ultimately be combined with other composition techniques before drawing conclusions on the material studied.

#### 3.3.3 Energy-Dispersive X-ray Spectroscopy (EDS)

EDS (or EDX) is another viable option for local composition analysis applying to both powders and electrodes (Scheme 1). EDS functions similarly to XPS, whereby X-rays are used to eject electrons from the shells of atoms. However, unlike XPS where the ejected electron is detected, EDS detects the X-rays released as an outer shell electron fills the electron–hole left behind by the ejected electron. EDS is not as insightful as XPS, because it does not measure the oxidation state of the elements detected and struggles more than XPS when measuring low atomic number elements. Additionally, due to the lower power of the X-ray beams used for EDS it can take long time periods to collect significant data.^155^ Furthermore, EDS is highly susceptible to inaccuracy when displaying relative concentrations.^156,157^ Despite EDS being heavily used (Figure 3A), due to its limitations it is often another supporting technique rather than a standalone characterization method.

The benefit of using EDS is that it enables composition scans to be performed while operating a SEM or TEM microscope, thus allowing for individual artifacts or larger areas to be analyzed in terms of composition rather than just observed as morphology. There are several ways of reporting EDS data, which include the overall spectrum that is similar in concept to the XPS survey scans, composition tables calculated from the said spectra, and elemental maps. Elemental maps are created by superimposing spectral maps on top of their respective microscopy images (Figure 7G–J). Scan times can vary significantly depending on the size of the area covered. For an elemental map to be statistically significant, a long enough scanning period must be used and multiple locations within the sample need to be validated to establish sample homogeneity. A key pitfall of MoS_2_ EDS is the fact that the Mo and S peaks overlap, making them difficult to distinguish.

### 3.4 Chemistry

#### 3.4.1 Raman Spectroscopy

Visible light–based characterization techniques offer another alternative for confirming MoS_2_ phase and quality. Unsurprisingly, Raman spectroscopy is the most widely used characterization technique within the surveyed literature (38.0% in Figure 3A), as it is simple, rapid, cost-effective, and applicable to a variety of sample types (Scheme 1). Raman spectroscopy involves inelastic light scattering typically following irradiation of the sample with a monochromatic laser. The laser wavelength can be varied from infrared to ultraviolet.^158^ The interaction between the incident photon and the sample material results in the excitation of electrons to a virtual energy state. Most often, this interaction is elastic and as the system relaxes the excited system transitions back to the ground state. In this case, a photon with the same energy and wavelength as the incident photon is emitted. This is referred to as Rayleigh scattering. However, in approximately one in a million cases^159^ the emitted photon can lose or gain energy associated with vibrational modes of the chemical bonds. The interaction between the incident photon and the material is thus inelastic. Subtracting the energy of the emitted photon from the incident laser gives the Raman shift (cm^–1^), which is plotted against intensity to produce a Raman spectrum.

**Figure 8:**
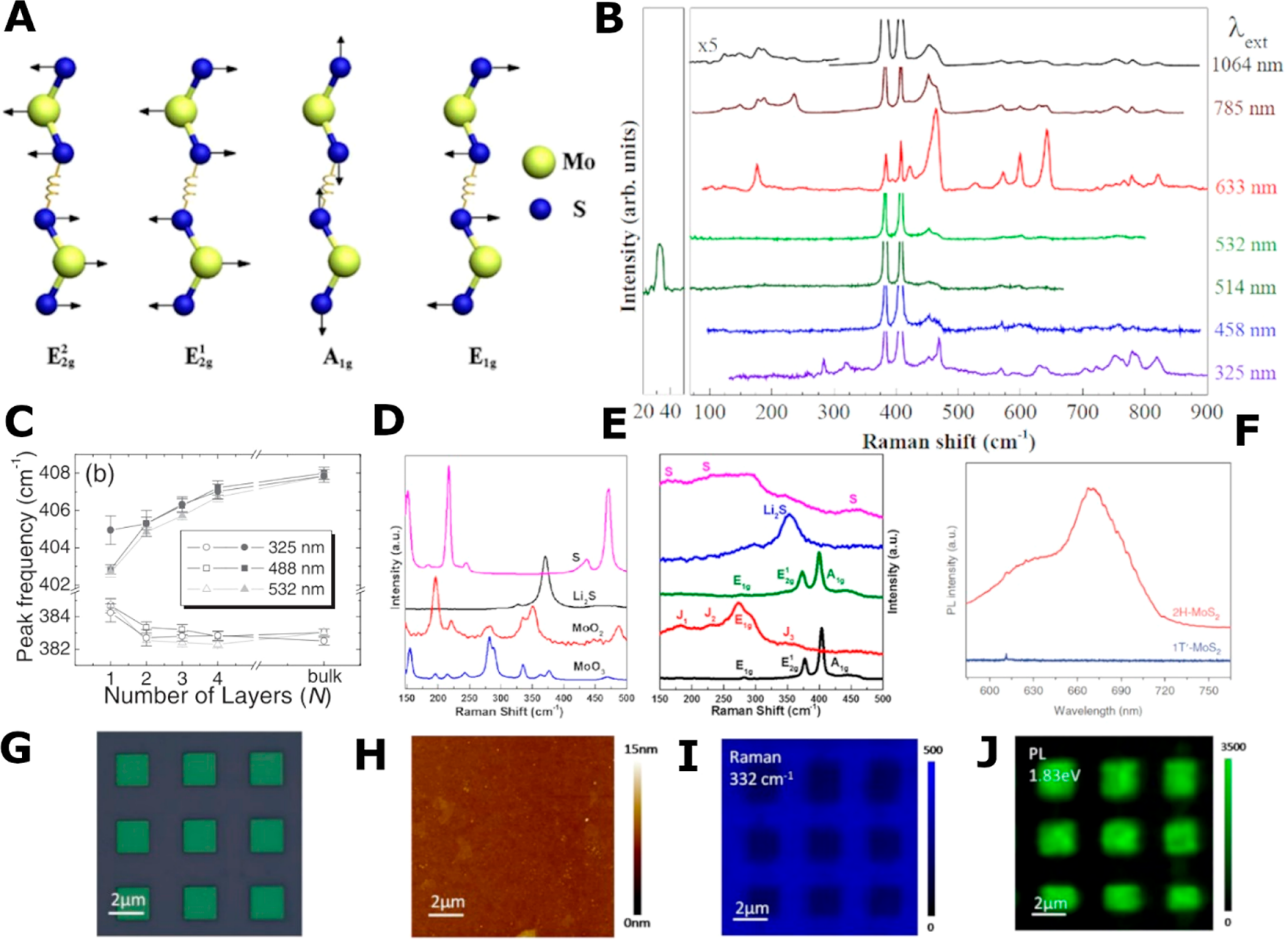
Chemistry techniques. (A) Illustration of the Raman phonon interactions on MoS_2_ atoms. Adapted with permission from (161). Copyright 2014 Nature. Raman: spectra of (B) top-down precursor MoS_2_ measured with different excitation wavelengths. Adapted with permission from ref (109). Copyright 2015 IOP Science. (C) Frequencies of *E*_2*g*_^1^ and A_1g_ varying with laser wavelength and layer number. Adapted with permission from ref (144). Copyright 2012 Wiley. (D) Typical MoS_2_ precursors used in bottom-up approaches (MoO_3_ and MoO_2_) and pure compounds expected to be formed during LIB discharge (Li_2_S and S), and (E) *ex situ* MoS_2_ electrode at various states during a discharge and recharge cycle in a LIB (black–pristine, red–discharged to 0.8 V, green–discharged to 0.8 V and charged to 3.0 V, blue–discharged to 0.05 V, magenta–discharged to 0.05 V and charged to 3.0 V). Adapted from ref (69). Copyright 2018 American Chemical Society. PL: spectra of (F) the distinction between 2H and 1T MoS_2_. Adapted with permission from ref (20). Copyright 2018 Nature. (G–J) Optical microscopy, AFM, J_3_ Raman mode, and PL of a masked MoS_2_ sample bombarded with Ar^+^ ions to cause controlled transition to 1T. The green boxes in (G) indicate the masking, which has subsequently been removed for (H–J) scans. Adapted from ref (131). Copyright 2017 American Chemical Society.

Peaks in the Raman spectra that are most typically used to study materials are those that arise from the scattering involving molecular or lattice vibrations (phonons) present in the sample. Each crystal structure will have characteristic phonon modes that are Raman active. Top-down precursor MoS_2_ has four off-resonance first order Raman active modes:^160^*E*_2*g*_^2^ (32 cm^1^),^109,160^*E*_1*g*_ (281 cm^–1^), *E*_2*g*_^1^ (379 cm^–1^), and *A*_1*g*_ (405 cm^–1^), which are depicted in Figure 8A. The *E*_2*g*_^2^ mode arises from the vibration in opposite directions of adjacent MoS_2_ layers in a multilayer structure. The *E*_1*g*_ mode involves the opposite vibration of S atoms in the plane of a single layer and in phase with the adjacent layer.^161^ The *E*_2*g*_^1^ symmetric mode involves the in-plane vibration of S atoms in one direction and the vibration of the Mo atom in the other. Adjacent layers are out of phase, vibrating in opposing directions. The *A*_1*g*_ peak arises from stretching of the S atoms out of plane, with both S atoms vibrating in opposite directions.^161^ Many more vibrational modes exist in MoS_2_, which are covered in detail in the works of Placidi et al.^109^

Typical Raman spectra of a top-down precursor MoS_2_ crystal are shown in Figure 8B, under a range of incident excitation wavelengths.^109^ The observed spectra clearly depend on the laser applied. The lasers used for Raman experiments within 73 different studies were found to fall in a rather narrow range of 512–532 nm (63%), 632–633 nm (14%) and 488 nm (3%). Most notably, 48% of papers use specifically the 532 nm wavelength, making this the easiest setup to directly contrast samples against the literature. Surprisingly, 19% of papers did not report the laser wavelength used in their experiments. Some Raman modes are extremely sensitive to the laser wavelength used,^107^ and again we encourage the detailed reporting of experimental conditions in the literature. Other aspects that alter Raman modes include the substrate, the temperature, and the presence of dopants or defects in the material.^160^

In Figure 8B, the only MoS_2_ features present throughout all seven incident wavelengths are the *E*_2*g*_^1^ and *A*_1*g*_ peaks, found at ∼379 and ∼405 cm^–1^ (Table SI 7). Generally, the intensity of *A*_1*g*_ is found to be greater than that of *E*_2*g*_^1^. The presence of both peaks identifies the material as 2H phase, albeit they can still be present in heterogeneous 2H/1T mixed phase MoS_2_ sample as well. Additionally, the *E*_2*g*_^1^ and *A*_1*g*_ peaks can also provide an indication of the layer thickness of few-layer exfoliated or bottom-up grown few-layer MoS_2_, based on the difference in wavenumber between these peaks (Δν = *A*_1*g*_ – *E*_2*g*_^1^).^160^ However, for mechanically exfoliated MoS_2_ crystals thicker than 5–6 layers or more (Figure 8C) the difference between the two peaks is similar as in top-down precursor MoS_2_ (Δν ∼ 26 cm^–1^).^109,160^

As the number of layers decreases from 5 to 1, Δν reduces as *E*_2*g*_^1^ upshifts and *A*_1*g*_ down-shifts in frequency. It is widely understood that the down-shift of the *A*_1*g*_ mode is due to the lack of suppression of atomic vibrations via stacking. For the *E*_2*g*_^1^ mode the upshift is believed to be due to stacking induced structural changes or the change in long-range Coulombic interlayer interactions.^109,160^ The upshift of *E*_2*g*_^1^ and down-shift of *A*_1*g*_ are reported in the literature as 2.2 and 4.1 cm^–1^, respectively, for mechanically exfoliated MoS_2_.^160^ Similarly, in the case of *n*-butyl-lithium exfoliated MoS_2_ there is a weak upshift in *E*_2*g*_^1^ with decreasing thickness, while on the contrary *A*_1*g*_ remains constant.^116^ Additionally, the relative intensity of *E*_2*g*_^1^ increases compared to *A*_1*g*_ as thickness increases. Eda et al. proposed that this occurs from weak interlayer coupling due to rotational stacking disorder following chemical exfoliation.^116^ Furthermore, the chemically exfoliated samples exhibited broader peaks relative to mechanically exfoliated ones^116^ perhaps due to the relatively greater defect density. The fwhm of a Raman peak is related to the lifetime of the phonon excitation. Sharper peaks result in smaller fwhm and indicate a greater sample crystallinity.

Raman spectroscopy is also an efficient and effective method to identify the phase of MoS_2_ samples. The transition from 2H to 1T or 1T’ MoS_2_ results in the disappearance of the key *E*_2*g*_^1^ and *A*_1*g*_ peaks, or a significant reduction in their intensity, alongside the appearance of three peaks *J*_1_ (147 cm^–1^), *J*_2_ (222 cm^–1^), and *J*_3_ (333 cm^–1^).^7^^,^^9^^,^^18^^,^^19^^,^^24^^,^^26^^,^^33^^,^^37^^,^^39^^,^^64^^,^^71^^,^^77^^,^^79^^,^^86^^,^^95^^,^^98^^,^^102^^,^^103^ In some cases, only the *E*_2*g*_^1^ peak will disappear and the *A*_1*g*_ peak remains in the 1T spectrum, since the *A*_1*g*_ mode can occur in single sheets as it involves only the vertical vibration of the S atoms (Figure 8A). The *E*_1*g*_ (281 cm^–1^) peak can be observed in both the 2H and 1T phases; however, it becomes more prominent in the 1T phase as seen in the discharge cycle of a LIB (Figure 8E). A further peak arising in the 1T phase, is located at a median 190 cm^–1^,^8^^,^^9^^,^^12^^,^^62^^,^^64^^,^^69^^,^^73^^,^^83^ and will be referred to as *Z*_1_. In energy application studies, *Z*_1_ is sometimes mistakenly confused with the *J*_1_ (147 cm^–1^) peak,^69^ as can be seen in Figure 8E. Thus, far, in application-oriented articles, the *Z*_1_ peak has only been suggested to arise from different MoS_2_ layers being present.^64^

With regard to the effects of changing the laser wavelength used, the work of Placidi et al. found that the *E*_2*g*_^2^ peak was only observed for the 514.5 nm wavelength^109^ (Figure 8B). Using UV light (325 nm) amplifies the spectral intensity of the characteristic bands, thus the *E*_1*g*_ (283 cm^–1^), *B*_2*g*_^1^, and higher frequency UV resonant peaks in the range 700–850 cm^–1^ are observed.^109^ Using resonant conditions, with lasers of wavelength 632 nm (exfoliated 2H MoS_2_ has a 1.9 eV direct bandgap), 786 nm and 1068 nm (2H top-down precursor MoS_2_ has a 1.29 eV indirect bandgap),^106^ results in the appearance of more modes.^109^ The lower frequency peaks (149.0, 188.1, 231.9, and 237.2 cm^–1^), normally associated with the 1T phase, are observed in 2H top-down precursor MoS_2_ irradiated with 785 nm or 1085 nm lasers due to indirect band gap resonance.^109^ Therefore, the 532 nm laser is optimal as a literature benchmark due to its wide usage (48%) and clear distinction between the 2H and 1T MoS_2_ phases. For the 532 nm laser wavelength the *J* and *Z*_1_ peaks are unique to the 1T phase.

For ease of phase identification, the full list of common MoS_2_ peaks used to distinguish between the 1T and 2H nanosheet phases is recorded in Table SI 7. Note that in most samples both a mix of 1T and 2H peaks will be observed as most samples are heterogeneous. Additionally, there is a large similarity between the 1T MoS_2_ and MoO_3_ peaks, which can appear due to high energy laser irradiation.^140^ However, it is often the Raman spectra of MoS_2_/carbon composites that are of most interest due to their application in energy systems. Raman spectroscopy of carbons is a vast and thoroughly investigated field of research.^158^ For composites, the most observed carbon modes are the D (∼1346 cm^–1^) and G (∼1587 cm^–1^) peaks, when measured with a 532 nm wavelength laser. The former is attributed to the breathing modes of sp^2^ atoms within rings and requires a defect or an edge state scattering for its activation, whereas the latter arises from the bond stretching of sp^2^ atom pairs in both rings and chains.^158^ This means that the D-mode will be observed in samples with small lateral dimensions, even if the material is pristine. In most cases, both peaks are observed as the majority of the sample is pristine with edge and point defects dispersed throughout.

Raman spectra can be collected as point or map scans.^121^ Point measurements offer fast localized sampling, yet the collection of multiple scans is required (>10) for a single point to achieve statistical significance and multiple locations need to be probed to guarantee sample homogeneity. Therefore, the employment of Raman mapping is a more statistically significant overall sample analysis method. Although Raman spectroscopy is considered a non-invasive technique, using an elevated laser power can result in local damage to the sample. Therefore, it is recommended to start at a low laser intensity and ramp up the power to reduce the signal/noise ratio, while ensuring the collected spectra do not change. Additionally, reporting Raman signatures of precursor materials such as MoO_3_^140^ (Figure 8D), or potential by-products, alongside the desired manufacturing products can be extremely beneficial to establish production routes.

#### 3.4.2 Photoluminescence (PL)

PL is an optical characterization technique which is applied to semi-conductors. Semi-conductors are excited by photons of higher energy than that of the bandgap of the material forming excited electrons and holes. Subsequently, the electrons and holes recombine emitting photons with energy typical of the band gap.^162^ Few-layer 2H MoS_2_ is semi-conducting and exhibits a clear PL spectrum, with two peaks around 630 and 670 nm, equivalent to 1.97 and 1.85 eV respectively, whereas metallic 1T MoS_2_ has no bandgap and thus does not produce PL^131^ (Figure 8F). In monolayer 2H spectra, higher intensity is observed than in quad, tri, or bilayers and is attributed to much slower electronic relaxation of 2H monolayer.^144^ This is because top-down precursor MoS_2_ is an indirect bandgap semi-conductor with a 1.29 eV bandgap, whereas 2H monolayer is a 1.9 eV direct bandgap semi-conductor.^106^

Like Raman spectroscopy, PL can be applied as a mapping technique with significant statistics, allowing 2H and 1T zones to be clearly identified in heterogeneous few-layer samples. In the work of Zhu et al., a few-layer sample of 2H MoS_2_ was selectively masked (Figure 8G), and subsequently bombarded weakly with Ar^+^ ions resulting in the transition from 2H to 1T in the exposed regions.^131^ To confirm this transition, a map scan of the same 100 μm^2^ area was undertaken with AFM, Raman spectroscopy, and PL (Figure 8H–J). The combination of these three techniques indicated that the sample was of uniform thickness (AFM), the exposed regions exhibited the *J*_3_ Raman peak, and no PL emission was detected, thus confirming the phase as 1T. The protected regions where the mask had been were strongly photoluminescent and had no *J*_3_ Raman peak, leading the authors to conclude that these regions remained 2H.

## 4 Discussion

A wide assortment of literature characterization techniques have been presented, which can be used to gain information about MoS_2_ and carbon composite architectures. Although, the list is by no means exhaustive, certain recommendations can be put forth concerning best practice for analyzing MoS_2_ based samples. As mentioned previously in this review, the statistical significance of the data obtained is of major importance. If data from a technique has a tendency toward low statistical significance, such as TEM, which only analyzes a minute area of the electrode sample, this must be taken into consideration. Ideally, low significance techniques should be performed multiple times, for instance carrying out multiple scans at the same point with Raman spectroscopy. If it is not possible to do so for practical reasons (*e.g.*, the time it would take to achieve significant statistics in TEM), the analysis carried out should only be considered as local validation rather than bulk material evidence. Lower statistically significant techniques (SEM, TEM, HRTEM, HAADF-STEM, AFM, SAED, and EDS) should be coupled with higher statistically significant techniques (HS-AFM, DLS, gas adsorption, XRD, XPS, TGA, Raman, or PL) to support relevant findings.

Although many articles claim the presence of heterogeneous 1T/2H MoS_2_ samples, there exist only six techniques that allow for the polymorphs of MoS_2_ to be identified. These techniques are HRTEM, HAADF-STEM, CS-AFM, XPS, Raman spectroscopy, and PL. High magnification techniques (HRTEM and HAADF-STEM) only allow for the classification of a miniscule amount of the sample,^152^ and often present both the 2H and 1T phases within a single nanosheet (Figure 4H and K). This makes them insufficient for classifying larger samples, although they may still be used to monitor nanoscale changes among *in situ* studies.^42,70,125^ CS-AFM cannot be presented alone as evidence for the presence of 1T MoS_2_, as conductivity alone is insufficient to determine the phase. However, in combination with other techniques, we encourage its use as a further source of confirmation. Similarly, PL alone cannot be used to confirm the presence of 1T phase MoS_2_, as 1T phase MoS_2_ is not semi-conducting and does not produce a PL signal. Nevertheless, the disappearance of PL signal occurs when 2H phase MoS_2_ transitions to 1T, or vice versa. This leaves only two available techniques that can be used to confirm the presence of 1T MoS_2_; XPS and Raman spectroscopy can both be used as proof of phase confirmation. Raman spectroscopy is the higher throughput lower cost option. However, to make the distinction unambiguous, both techniques should be applied together where possible.

For energy applications, to fully characterize large 3D structures such as electrodes, the use of multiple characterization perspectives is necessary. Based on Figure 3A, a large fraction of MoS_2_ research articles contain SEM, TEM, XRD, XPS, and/or Raman spectroscopy (>20%). However, TEM can be discarded due to its low statistical significance, low throughput, high cost, and its inability to image thick structures. Ideally, the four remaining techniques (SEM, XRD, XPS, and Raman spectroscopy) are presented together as the base sample “fingerprint”, as they allow for broad comparison with the literature. Moreover, between these four techniques:

- all the characterization categories are covered (morphology, crystal structure, composition, and chemistry);
- the techniques are widely available and low cost;
- the operating time and skill to entry are low;
- the 1T/2H phase can be established (XPS and Raman spectroscopy);
- a statistically significant macro analysis is carried out.

Nevertheless, for material synthesis studies with an application objective, in addition to the base fingerprint the use of product validation of the synthesis powders, solutions, or dispersions is encouraged (Scheme 1). For morphology, in addition to SEM, the combination of TEM, AFM/HS-AFM, and DLS makes it possible for lateral size distributions to be quantified from three separate angles. DLS can, however, only be applied to solutions and dispersions. Sample thickness can be measured by both AFM and TEM, although both techniques struggle with statistical representation and the use of HS-AFM is therefore encouraged if available. Regarding crystal structure, HRTEM and SAED can be used alongside XRD to gain further local crystallographic information. For composition, EDS and TGA can be applied as additional corroboration of XPS findings. Finally, additional specific techniques can be used for *in situ* studies of synthesis routes or device applications (TEM, SAED, XRD, and Raman spectroscopy). However, *in situ* studies should not overly rely on a single detailed characterization technique and should use further *ex situ* characterization to corroborate findings.

Characterization data should make use of good reference samples. Good characterization references can be the inclusion of pristine top-down precursor MoS_2_, bottom-up reactants, and suspected by-products as a comparison. In production routes, this implies providing characterization at multiple steps within the process, so that the overall mechanism can be understood. Similarly, if changes in the process are investigated, such as altering reactant amounts or operating conditions, characterization of the different products should be thoroughly disseminated. For energy application purposes, in the simplest of forms this can be interpreted as measurements before and after testing, regardless of the duration of the testing. Alternatively, more detailed information can be provided with *ex-situ*, *in situ*, or *operando* characterization where possible.^41^^,^^45^^,^^52^^,^^66^^,^^67^^,^^69−71^^,^^143^

Within the published literature, more experimental detail should be provided regarding characterization since 21% of manually surveyed articles omit identifying the XRD source and 19% do not report the Raman laser wavelength. Furthermore, the number of scans collected for XPS and Raman point scans and overall DLS scans should be provided, as they influence the statistical significance. Within microscopy-based techniques, such as SEM, TEM, HRTEM, HAADF-STEM, AFM, EDS, and SAED, observation and measurement counts need to be reported clearly. Additionally, microscopy-based techniques should follow a more thorough supporting information sample dissection protocol to provide a more holistic sample overview, rather than offering the single best micrograph or scan taken. Finally, the underlying assumptions of characterization techniques need to be included more regularly, for instance TGA product assumptions.

Despite the advancements in MoS_2_ characterization, there are several topics which remain unclear and disputed within the scientific community. As mentioned earlier, the exact thickness of a single MoS_2_ nanosheet measured with AFM is unresolved. The reported values encompass a range of 0.7 nm −1.2 nm.^6,8−10^^,^^15^^,^^20^^,^^21^^,^^24−26^^,^^28^^,^^29^^,^^57^^,^^77^^,^^87^^,^^88^ However, the degree to which this spread is caused by the manufacturing method,^116^ experimental uncertainty, substrate material used, or instrument calibration is unclear. Eda et al. reported that mechanically exfoliated single nanosheets are approximately 0.7 nm thick due to their pristine surfaces, whereas the more aggressive *n*-butyl-lithium chemical approach results in corrugated nanosheet surfaces resulting in a thickness of 1.0 nm −1.2 nm.^116^ Further research has not explored the effects on nanosheet thickness of the full range of manufacturing techniques, substrates, and AFM instruments.

With regards to the down-shift in XPS binding energies observed between 2H and 1T MoS_2_ (Figure 7E), even though in most instances the down-shift energies are close to 0.9 eV, there appear to be six papers which all report a smaller down-shift of 0.4–0.5 eV for 1T or 1T’ MoS_2_.^10,26,34,93,101,122^ Tiwari et al. report the Mo 3d peak position as a gradual down-shift with cycling of their MoS_2_ composite in a supercapacitor, achieving a down-shift of ∼0.42 eV after 2500 cycles.^101^ The other five papers report a 0.5 eV XPS down-shift as a result of material synthesis^10,26,34,101,122^ and cite five other articles to support their 0.5 eV shift.^116,163−166^ Four of the synthesis papers use hydrothermal methods,^10,34,101,122^ and one relies on supercritical CO_2_ coupled with liquid exfoliation.^26^ However, the cited articles never report or mention a shift in binding energies. Yu et al.^163^ is the only cited paper that, in turn, cites theoretical works suggesting an excitonic binding energy of 0.4–0.8 eV in MoS_2_. Of the six papers mentioned earlier that claim a 0.4–0.5 eV down-shift, two are by the same author Cai et al.^10,122^ Currently, the source of this deviation from the generally accepted ∼0.9 eV binding energy down-shift remains unclear, and a study on production route influence on XPS Mo peak positioning would be beneficial.

Although, the Raman spectroscopy *J* peaks are commonly observed in experimental spectra within application-oriented articles, there is a lack of understanding of their origin within the energy field. Typically, the *J* peaks are utilized to classify MoS_2_ samples as 1T. However, the *J* peaks are not unique to 1T MoS_2_, as they have also been observed under irradiation with 785 and 1064 nm lasers in top-down precursor MoS_2_ (Figure 8B), which is expected to be 2H.^109^ The observation of *J* peaks at laser wavelengths 514–633 nm^7^^,^^9^^,^^18^^,^^19^^,^^24^^,^^26^^,^^33^^,^^37^^,^^39^^,^^64^^,^^71^^,^^77^^,^^79^^,^^86^^,^^90^^,^^98^^,^^102^^,^^103^ might arise due to the transition from an indirect bandgap semi-conductor in top-down precursor MoS_2_ (*i.e.*, 2H MoS_2_) to a metallic conductor 1T MoS_2_, whereby resonance can now occur at any incident wavelength. Hence, reporting of experimental conditions becomes crucial to enable accurate phase confirmation via Raman spectroscopy. Further studies, utilizing a broad range of laser wavelengths on 1T MoS_2_ samples, would solidify the understanding of Raman spectroscopy for MoS_2_ phase confirmation.

Despite 1T MoS_2_ being classified as long ago as 1992,^127^ and the existence of multiple macroscopic 1T synthesis routes,^7−12^^,^^14^^,^^18−21^^,^^24−26^^,^^28^^,^^33^^,^^34^^,^^37^^,^^39^^,^^52^^,^^62^^,^^75^^,^^77^^,^^83^^,^^95^^,^^97^^,^^102^^,^^103^^,^^116−118^^,^^121^^,^^126^^,^^127^^,^^131^ the origin of the transition from semi-conductor to metal MoS_2_ remains unclear. It is postulated that induced strain is the root cause of the phase transition in all manufacturing methods.^106^ Lithiation based transition has been most investigated due to the interest in MoS_2_ performance in LIBs. However, *in situ* lithiation studies have not provided further proof regarding the mechanism of this transition. The most detailed *in situ* STEM MoS_2_ transition study to date, using low quantity Re doped MoS_2_, revealed the generation of Mo–Mo compression at elevated temperatures.^125^ Three MoS_2_ rings compress in a strip, referred to as the α-phase. Where two α-phases intersect, the triangular region between them builds up a negative charge due to the STEM electron beam. In a rapid release of tension, the entrapped area glides away from the α-phase boundaries resulting in the formation of a localized 1T phase. However, the cause of the formation of the α-phase is unknown, and the observed phase transformations were initiated at locations where Re substituted Mo in the MoS_2_ lattice. Whether this same mechanism is at play in hydrothermal production or LIB operation is unclear.

Furthermore, the distorted 1T’ polymorph of MoS_2_ has become a disputed topic regarding conductivity. Density of state calculations by Zhang et al. have predicted the distorted 1T’ MoS_2_ to be semi-conducting with a similar bandgap to monolayer 2H MoS_2_.^69^ However, experimental PL spectra from Yu et al. show no PL emission from 1T’ MoS_2_ (Figure 8F). Hence, the authors claim their sample to be metallic.^20^ By annealing their 1T’ sample, it reverts to 2H monolayer with distinct PL peaks. Yu et al. used STEM, XPS and X-ray absorption fine structure (XAFS) to further support their characterization. Except for STEM, none of the other techniques can distinguish between 1T and 1T’. As previously mentioned, STEM is not statistically significant to solely confirm the entirety of the sample as 1T’, thus meaning that most of the sample could have been metallic 1T rather than semi-conducting 1T’. Further investigation is required to confirm whether the theoretical work by Zhang et al. or the experimental work by Yu et al. is correct on the conductive nature of 1T’ MoS_2_. Identifying a characterization methodology to distinguish 1T and 1T’ MoS_2_ phases in large amounts could clarify the situation.

Lastly, LIB MoS_2_ studies, which form a large part of the surveyed MoS_2_ research (13.3% of classified energy application research), struggle to agree on the storage mechanism experienced by MoS_2_ electrodes. During the first lithiation process, it is widely agreed that MoS_2_ initially forms the intercalation compound Li_*x*_MoS_2_, and upon further lithiation breaks into the decomposition products Mo and LiS_2_. However, there are two schools of thought regarding the first delithiation step. Some postulate that Li_2_S converts into elemental S_8_, and the Mo remains chemically inactive. This implies that only the sulfur operates in the storage mechanism, leading to the degradation of the cell with cycling.^41,66−70^ Others believe that Li_2_S and Mo react upon delithiation to produce nanosized domains of MoS_2_.^37,43,45^ Because of the small size of the reformed MoS_2_ it cannot be detected with XRD. Consequently, the reaction in the following cycles is reversible and nano–sized MoS_2_ degrades into Li_2_S and reforms on every cycle. Ultimately, two contradictions arise from the dispute. The irreversible school of thought relies on commercial MoS_2_ and larger scale characterization techniques and thus it is possible for them to omit small–scale changes. On the other hand, the reversible understanding relies on synthesis routes producing MoS_2_/CNT or MoS_2_/graphene composites, and small-scale characterization techniques such as TEM/HRTEM, which cannot capture the full-scale change of the electrode. A combined study, focusing on using a wide range of characterization techniques *ex situ* and *in situ*, on both commercial and MoS_2_ composite products could clarify the MoS_2_ LIB mechanism.

## 5 Conclusion

Overall, MoS_2_ has a unique characterization fingerprint, which has been distilled from a variety of characterization perspectives. We have proposed the use of the most accessible techniques (SEM, XRD, XPS, and Raman spectroscopy) as the base fingerprint of MoS_2_ energy application-oriented characterization, to enable the ease of future MoS_2_ based sample comparison. Similarly, we have suggested the use of XPS and Raman spectroscopy to classify 1T MoS_2_ phase comparably to other literature studies. Additionally, users can find suitable supplementary characterization techniques from the wide assortment presented within this review. Furthermore, less widely available specialist techniques can be used for detailed understanding. Within this review, suggestions have been put forward on how best to combine characterization techniques to provide multiple sources of confirmation, account for technique setbacks, and consider statistical significance by critically evaluating the sample selection analyzed. We also guide the reader on including the most valuable data for future MoS_2_ experimental and computational analysis, by advocating the reporting of experimental conditions, the provision of beneficial reference samples, and highlighting the importance of measurement count reporting.

Ultimately, MoS_2_ is a material of significant interest in the energy storage and energy conversion fields, with energy application publications increasing and including multiple battery chemistries, supercapacitors, and hydrogen evolution catalysis. This is a result of the wide range of MoS_2_ production routes, which allow for great control of the material morphology, properties, and composite architectures. However, as has been highlighted throughout this review, there is a lack of MoS_2_ characterization standard to be addressed throughout the application performance literature. Nevertheless, MoS_2_ faces many exciting unresolved challenges in characterization for energy applications. These include the isolation of pure 1T MoS_2_, understanding the 2H to 1T phase transition, agreeing on the LIB degradation mechanism of MoS_2_, resolving the MoS_2_ nanosheet thickness disparity within AFM, improved understanding within the energy field of the MoS_2_ Raman peak locations and nomenclature, and identifying the sources of the variance in the literature for the XPS 1T phase downshift.

## Supporting Information Available

Manual and Web of Science API methods for literature publication data, including survey comparison figures, variance calculations, manual query search tables, classification field term tables (energy application, production, and classification), and characterization technique key value tables (XPS and Raman spectroscopy) (PDF)

## Vocabulary

Nanosheet: Atoms or molecules arranged in a sheet-like structure, exhibiting both length and width (typically >10s nm), but only a nanoscale thickness (on the order of a few nm). Although the lateral dimensions can vary significantly from nanometers to micrometers, the key factor is the nanometer thickness of the sheet. Sheets can be monoelemental, such as graphene or phospherene, or compounds such as MoS_2_, MoSe_2_, MoTe_2_, TiS_2_, WS_2_, FeSe_2_, Bi_2_Te_3_, and Sb_2_Te_3_.
Layered material: A material including a number of individual nanosheets. Nanosheets are stacked vertically on top of each other like a deck of cards. This results in the generic flake-like structure of layered materials. Adjacent sheets are held together by van der Waals forces.
Exfoliation: The process of separating individual nanosheets from flakes of a layered material.
Top-down precursor MoS_2_: A flake-like structure of MoS_2_, containing a multitude of nanosheets. This is the material form achieved by refining molybdenite ore or synthetically produced by laboratory suppliers. The term top-down precursor MoS_2_ or commercial MoS_2_ is used within the review to avoid the ambiguous term “bulk” MoS_2_, which is often used in the literature. This nomenclature is introduced to distinguish between bulk material characterization techniques and the MoS_2_ nanosheet synthesis precursor material.
Top-down synthesis: A manufacturing process for MoS_2_ nanosheets or MoS_2_/carbon composites, where top-down precursor MoS_2_ is used as the initial starting material. Nanosheets are produced by exfoliating top-down precursor MoS_2_.
Bottom-up synthesis: A manufacturing process that involves the buildup of material from the bottom: atom-by-atom, molecule-by-molecule, or cluster-by-cluster.
Statistical significance: A large enough segment of the material sample/data/literature has been analyzed to justify claims that the sample is homogeneous or heterogeneous throughout, the data is not an experimental outlier, or the topic makes up a significant part of the available literature. Bulk material characterization allows statistical significance to be achieved with a single measurement, whereas localized measurements require a large measurement distribution.
