# Supplementary material for: Ex Situ Characterization of 1T/2H MoS2 and Their Carbon Composites for Energy Applications, a Review
Source: ACS Nano. 2023 Mar 16;17(6):5163–86. doi: 10.1021/acsnano.2c08913 (PMC10062033; doi:10.1021/acsnano.2c08913)
Supplement: Supplementary file 1 — nn2c08913_si_001.pdf [file nn2c08913_si_001.pdf]

Supporting Information

ACS Nano

February 17th, 2023

Title: *Ex situ* Characterization of 1T/2H MoS_2 and Their Carbon Composites for Energy Applications, a Review

Authors: Alexandar D. Marinov[(1)], Laura Bravo Priegue, Ami R. Shah[(1)], Thomas S. Miller[(1)], Christopher A. Howard[(2)], Gareth Hinds[(3)], Paul R. Shearing[(1)], Patrick L. Cullen[(4) ǂ], and Dan J. L. Brett[(1) ǂ]

1. Electrochemical Innovation Laboratory (EIL), Department of Chemical Engineering, University College London (UCL), Gower Street, London, WC1E 6BT, UK
2. Department of Physics & Astronomy, University College London (UCL), Gower Street, London, WC1E 6BT, UK
3. National Physical Laboratory, Hampton Road, Teddington, TW11 0LW, UK
4. School of Engineering and Materials Science, Queen Mary University of London, Mile End Road, London, E1 4NS, UK

ǂ Corresponding author(s)

Supporting Information

1. Manual Literature Search Engine Statistics

Using the official search engines Scopus, Science Direct, and PubMed the annual match (hit) count for the term 'MoS2' was manually exported using the search queries presented in Table SI 1. This allows for comparison between the search engines and the Web of Science API method from section 3 (Figure SI 1).

2. Manual Article Categorization

One hundred energy application-oriented research articles (review papers and other article types were excluded) were selected. An attempt was made to include a wide variety of possible energy applications to the best of the authors' knowledge. All the papers were published in the year range 2011-2022. The 100 papers selected were some of the first search results in Google Scholar or Web of Science for the relevant topic (e.g. MoS_2 LIB or MoS_2 HER). Therefore, the 100 papers selected do not represent the entirety of the literature. However, it enabled the authors to identify classification categories for MoS_2 research.

Generally, research articles contain three distinct sections. Firstly, synthesis (e.g. hydrothermal) where a production method or a variation of a literature method is used to synthesize a nanoscale morphology MoS_2. Papers that do not include such a section, utilize top-down precursor MoS_2 throughout their studies. Secondly, characterization where a variety of techniques are applied on the synthesis product. The detail of this section greatly varies between studies. Finally, an application performance section. The only research articles that do not follow the outlined recipe, focus solely on the development of synthesis methods, furthering characterization techniques, or understanding the operating mechanism of applications through *in-situ* characterization.

For each of the papers considered, the energy application (Figure SI 2), production method (generic production in Figure 1B and 1T phase MoS_2 synthesis in Figure 1C) and characterization techniques employed (Figure SI 3) were logged manually. Once all the data was gathered, categories could be identified. The reasoning behind the establishment of the various categories is provided below.

Energy Application: The application of the paper was established based on which application performance results were most heavily favored in the title, abstract, figures, text body, and conclusion (Figure SI 2). Hydrogen evolution catalysis was divided into purely electrochemical (HER) and photoelectrochemical HER (photo-HER). Battery chemistries involved lithium-ion batteries (LIB), sodium-ion batteries (SIB), potassium-ion batteries (KIB), and lithium-sulphur batteries (LiS). Additionally, supercapacitors and photoelectrochemical catalysis (PEC) were included.

Production: The top-down and bottom-up production routes classifications already existed in the literature[(1)]. Within top-down synthesis the overall categories of mechanical exfoliation, sputtering, and liquid phase-assisted exfoliation exist. Liquid phase exfoliation can be expanded into purely liquid phase exfoliation and intercalation assisted liquid phase exfoliation. Intercalation exfoliation includes organo-alkali solvents such as n-butyl-lithium and sodium-napthalenide, electrochemical intercalation, and the metal-ammonia method (Figure 2B). On the other hand, bottom-up methods include hydrothermal, solvothermal, chemical vapor deposition (CVD), and solid state reactions. Hydrothermal and solvothermal methods are distinguished by their use of solvent. CVD grows MoS_2 nanosheets from elemental Mo and S powders. Solid state reactions are distinguished from CVD, by mixing their powder reactants together prior to the high temperature treatment. 'Multiple' encompasses studies which use any combination of the above mentioned synthesis methods. Commercial MoS_2 refers to papers which directly purchase top-down precursor MoS_2 and use it within their studies without employing any synthesis steps or further treatment.

Characterization: When surveying any energy application-oriented research article, all the characterization techniques employed were recorded. Multiple techniques could be present within a single paper. At the end of the survey, techniques were grouped together to form larger objective focused characterization categories: morphology, crystal structure, composition, chemistry, and other. Only the commonly applied techniques were presented in Figure SI 3 and discussed throughout the review.

3. Web of Science API method

The Web of Science Expanded API was used throughout the study. Only articles of type research article were utilized within this statistical analysis. Other article types, such as review papers were neglected. Research articles were downloaded using matches for the 'MoS2' search term (Figure SI 4). Web of Science was utilized, because it enabled access to the largest pool of MoS_2 research articles out of any literature database available (Figure SI 1), with 40,422 research articles available from 1990 to 2022. However, to keep the review relevant and allow for text scanning to take place, only articles in the year range 2011 to 2022 were considered for categorical classification (energy application, production, and characterization), as articles published before the rise of the internet might not be in a computer readable format. Thus, the

total number of papers considered for categorical classification from 2011 to 2022 was 37,347. Out of these, 0.7 % were discarded as the text (title, abstract, and/or keywords) was inaccessible and classification could not take place.

Before classification could take place, papers were pre-processed to eliminate unnecessary elements from the text. Titles, abstracts, and keywords were pre-processed using the python Natural Language Processing library (NLTK). This procedure involved lower-casing all text, removing punctuation, and lemmatization (removing suffixes and prefixes to reduce a word to its most basic form, which is known as the lemma). Three separate and independent classifications were carried out (Figure SI 4). These focused on energy application, production methods, and characterization techniques used. For each of the classifications, text scanning was conducted on the title, abstract, and keywords. A selection of field terms composed of the field word and their synonyms for each of the classification sub-categories were considered as displayed in Tables SI 2-4. Each of the field terms were used for keyword-matching and keyword-counting using Regular Expressions (RegEx) in Python. RegEx are combinations of characters used to construct complex find and/or replace searches.

Therefore, within a single classification (e.g. energy application) it was possible to search and add-up the number of appearances of each of the field terms in the text. As a result, a count was obtained for each sub-category for a given paper. In the case of a tie (for instance a paper researching both LIB and HER with equal counts of both field terms), the paper was randomly allocated to one of the tied sub-categories. This ensured that for energy application and production methods classification, each paper was only counted once towards a single sub-category. In the case of characterization technique classification, each paper could be counted towards multiple sub-categories, as papers rightfully use multiple characterization techniques.

However, not all words are of equal importance. The terms that form the title and list of keywords, tend to be very carefully selected and relevant to the topic of the paper. Meanwhile, words in the abstract do not necessarily carry the same significance. To account for this, a weighted contribution was utilized, whereby matches in the title and keywords were scaled by a factor of two, whereas matches in the abstract were counted as unity. The counts contributing towards each sub-category (through field terms) were aggregated, and the dominating sub-category was assigned as the paper application or production method.

To verify the accuracy of the Web of Science API method, manual quality assurance of a subset of the classified data was carried out. This was done in an iterative approach, adding and removing fields and synonyms based on review feedback, and operating the algorithm again. Several iterations were undertaken until the final classification field terms presented in Tables 2-4 were established. We were unable to establish the production method classification field terms and synonyms to a high enough standard validated by human control, however we have included the terms used for future reference (Table SI 3).

In the case of characterization technique classification, additional care had to be taken as classification is not unique (each article can match to multiple sub-categories), and there is a large degree of overlap between several sub-category synonyms. The synonyms ‘electron microscopy’, ‘transmission electron microscopy’, and ‘high resolution transmission electron microscopy’ corresponding to the sub-categories SEM, TEM, and HRTEM, respectively (Table SI 4), overlap

on the phrase ‘electron microscopy’. If ‘electron microscopy’ is preceded by the term ‘transmission’ the correct classification ought to be TEM. However, since classification is not unique, it registers towards both sub-categories (SEM and TEM). Similarly, acronyms also experience this issue. For instance, the term ‘tem’ is part of ‘hr tem’, and would be counted towards both the TEM and HRTEM sub-categories. Table SI 4 highlights problematic synonyms in bold. To address this issue, a RegEx protocol was employed restricting text match segments to single unique sub-categories, where the largest part of the term scanned is found. Hence, for the text “…high resolution transmission electron microscopy was used to elaborate the findings on…” the major part of the text matches mostly the HRTEM synonym (Table SI 4), and will be classified as the sub-category HRTEM.

Several other issues were also encountered within the Web of Science API method and have been flagged for future reference. Removing punctuation such as ‘-’ and lemmatization resulted in grammatically incorrect spelling of UV-vis and XRD/XPS/EDS, including terms such as ‘uv vi’ and ‘x ray’. These grammatically incorrect synonyms have been added to Table SI 4 as they are crucial for an accurate classification count. Utilizing a single search term for the Web of Science API (‘MoS2’). It is possible to encounter papers, which do not focus on MoS_2 but use it as a ‘buzz word’ to attract attention to their own work on another TMD, graphene, or other 2D material because of the large interest surrounding MoS_2. Therefore, we believe in future API methods, articles should be prefiltered by counting occurrences of the search term itself.

Regarding production methods classification the lack of reporting within the title, keywords, and abstract made it difficult for production methods to be counted correctly. The only production sub-categories often mentioned in the accessible text are hydrothermal, solvothermal, and CVD. Furthermore, commercial MoS_2 is the initial precursor for top-down production methods or a common application comparison. Hence, it appears often in the text even if a different production method is actively applied.

Therefore, we would encourage for future research articles to make use of search engine optimization (SEO), by utilizing high value and relevant to the research work terms, which can be easily picked up by literature search engines and APIs. For energy application-oriented research, this consists of including the production method and energy application within the title and keywords, and including a list of the main characterization techniques within the abstract. In the case of solely working on synthesis, the production method should be found in the title and keywords. For purely characterization work involving MoS_2, the dominant technique should appear in the title and keywords, with other techniques presented in the abstract. For *in-situ* characterization regardless of whether focusing on application mechanism, synthesis, or properties, the word *in-situ* along with the characterization technique should appear in the title alongside the scope (application mechanism, synthesis, or property). In the case synthesis is not the focus of the *in-situ* study, the production route should be at least mentioned in the abstract. Finally, we believe the future development of a material science anthology allowing for computational methods to achieve more than just classification through text scanning, could be incredibly beneficial for the field.

4. Survey Comparison and Variance

A variance approach was carried out on the manual 100 energy application-oriented and API method papers classified for energy application and production. Table SI 5 uses the Equation SI 1 to find the total variance (for the entire year range considered) for each of the classifications methods where a paper can only fall within a single sub-category (application and production). In the case a paper classification can count towards multiple sub-categories (characterization) variance is not applicable. Equation SI 1 is applied throughout, where σ is the total variance, N is the total number of sub-categories, i represents the sub-categories, δ is the average count across all the sub-categories considered, and α_i is the total count for specific sub-category i.

Equation SI 1: $$\sigma = \sum_i^N \alpha_i - \delta$$

Analyzing the manual energy application classification variance, it is heavily uneven with a bias towards LIBs (Table SI 5). Similarly, the manual classification of production methods is heavily bias towards hydrothermal production, as favored by LIB articles. However, the purpose of the manual survey was only to establish a wide range of sub-categories for energy applications, production routes, and characterization techniques. In fact, the total variance calculations on the API method data, indicated that the MoS_2 literature overall is heavily unequally distributed with bias towards HER.

Comparing the manual method and API method, both indicate a strong presence of LIB and HER. Where the manual method fails, is that it does not have a large enough sample size to see the true presence of HER within the field and omits the importance of supercapacitors. However, when it comes to characterization, the API method can only locate techniques found in the title, keywords, or abstract and therefore fails to observe supporting characterization techniques. For instance, Figure SI 3 indicates that HRTEM is often found alongside TEM as they are carried out in the same microscope, which Figure 3A fails to agree on as HRTEM is not mentioned as often in paper abstracts. Similarly, the case with EDS and SAED that are generally supporting techniques and not key findings.

Figures:

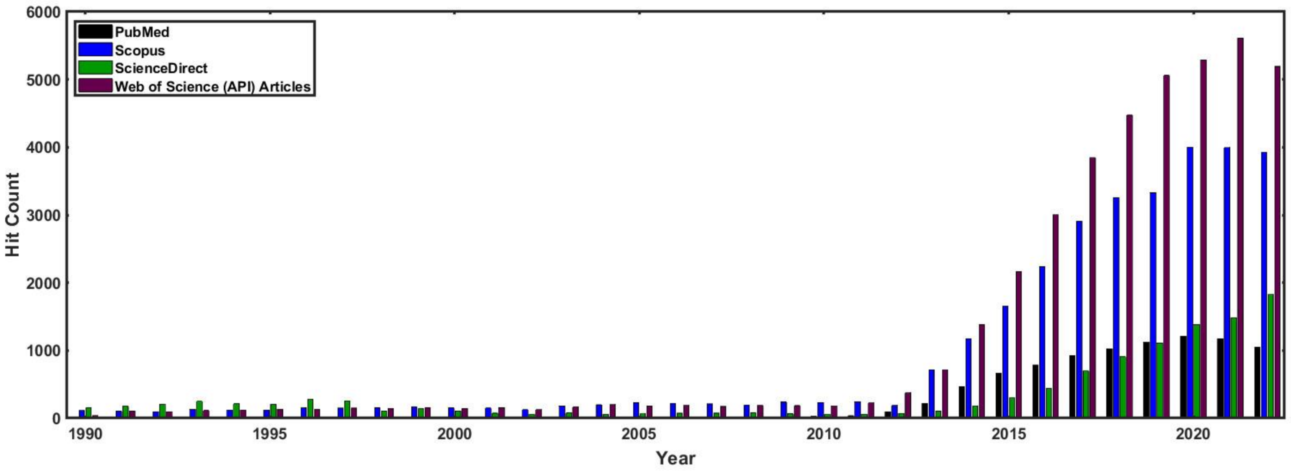

Figure SI 1 – MoS_2 search hits using different literature search engines or Web of Science API. Data sources and date data taken on are listed in Table SI 1.

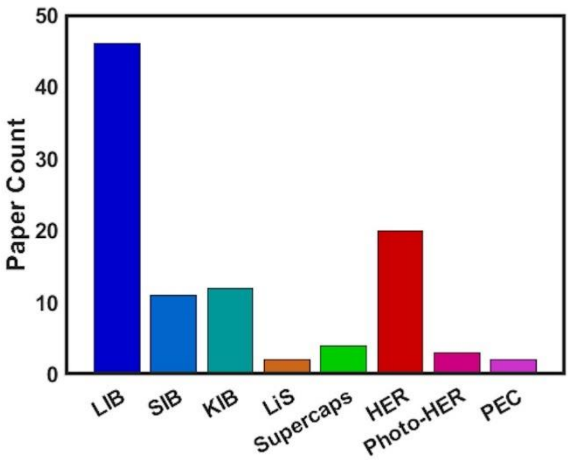

Figure SI 2 – Manual classification of 100 energy application papers for energy applications.

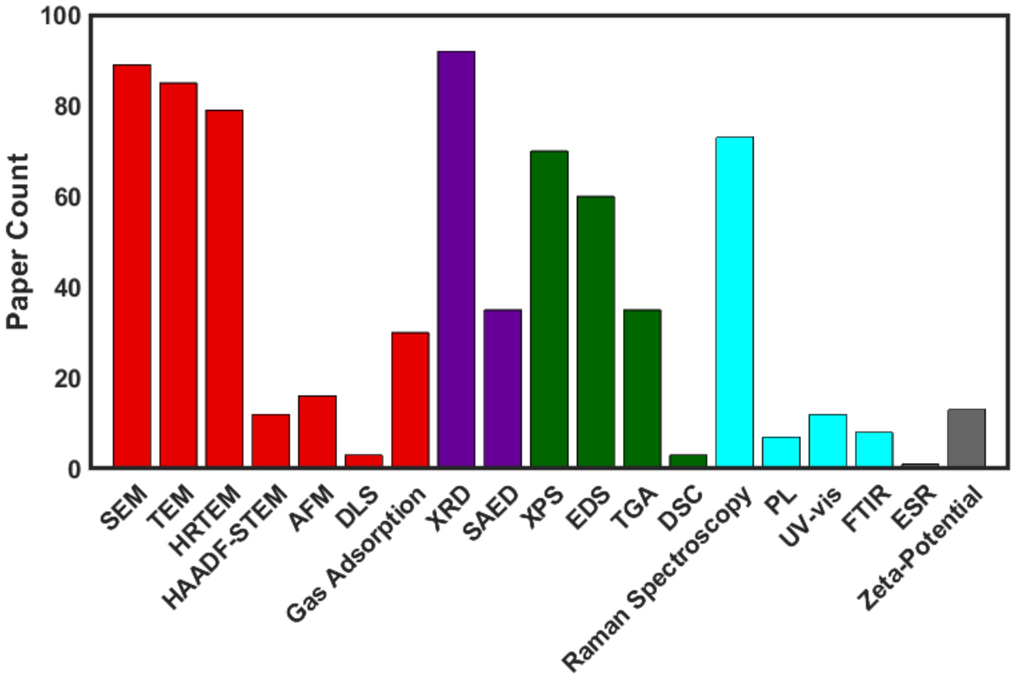

Figure SI 3 – Manual classification of 100 energy application papers for characterization techniques.

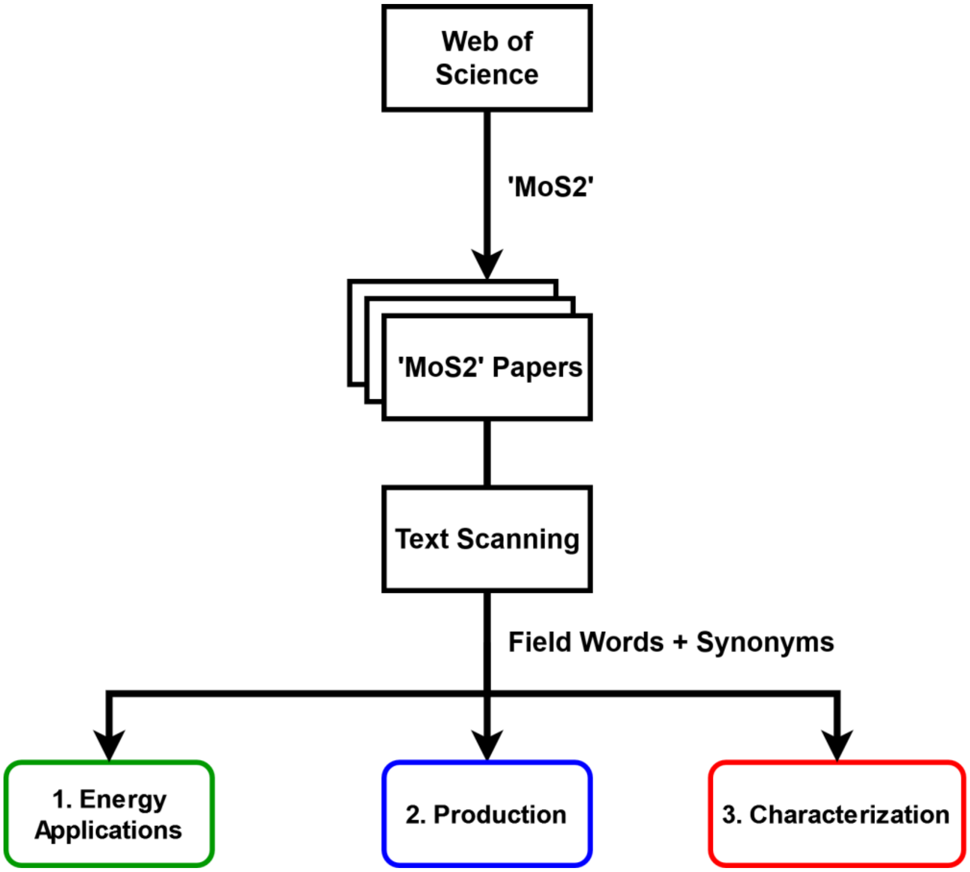

Figure SI 4 – Web of Science API and text scanning classification method schematic.

Tables

Table SI 1: Manual search engine queries and API used to gather scan hits for search term 'MoS2' from 1990 to 2022. Data in Figure SI 1 is used to compare manual searches to API.

Search Engine	Date Carried Out	Hit Count	Query Link
Scopus	11/11/2022	31,087	https://bit.ly/3VBY6Wp
Science Direct	13/11/2022	11,349	https://bit.ly/3GXGyQL
Web of Science	11/11/2022	43,476	https://bit.ly/3HeYExR
PubMed	11/11/2022	8,933	https://bit.ly/3ON2wYl
Web of Science (API)	30/11/2022	40,422	

Table SI 2 – API classification algorithm synonyms for category: energy application.

Field Word	Synonyms
LIB	lib, libs, lithium ion battery, lithium ion batteries, lithium cell, lithium ion cell, lithium metal battery, lithium metal batteries, lithium metal cell, li ion battery, li ion batteries, li cell, li ion cell, li metal battery, li metal batteries, li metal cell
SIB	sib, sibs, sodium ion battery, sodium ion batteries, sodium cell, sodium ion cell, sodium metal battery, sodium metal batteries, sodium metal cell, na ion battery, na ion batteries, na cell, na ion cell, na metal battery, na metal batteries, na metal cell
KIB	kib, kibs, potassium ion battery, potassium ion batteries, potassium cell, potassium ion cell, potassium metal battery, potassium metal batteries, potassium metal cell, k ion battery, k ion batteries, k cell, k ion cell, k metal battery, k metal batteries, k metal cell
MIB	magnesium ion battery, magnesium ion batteries, magnesium cell, magnesium ion cell, magnesium metal battery, magnesium metal batteries, magnesium metal cell, mg ion battery, mg ion batteries, mg cell, mg ion cell, mg metal battery, mg metal batteries, mg metal cell
LiS	lithium sulphur battery, lithium sulphur batteries, lithium sulphur cell, lithium sulphur metal battery, lithium sulphur metal batteries, lithium sulphur metal cell, lithium sulfur battery, lithium sulfur batteries, lithium sulfur cell, lithium sulfur metal battery, lithium sulfur metal batteries, lithium sulfur metal cell, li sulphur battery, li sulphur batteries, li sulphur cell, li sulphur metal battery, li sulphur metal batteries, li sulphur metal cell, li sulfur battery, li sulfur batteries, li sulfur cell, li sulfur metal battery, li sulfur metal batteries, li sulfur metal cell
Li-Air	lithium air battery, lithium air batteries, lithium air cell, lithium oxygen battery, lithium oxygen batteries, lithium oxygen cell, li air battery, li air batteries, li air cell, li oxygen battery, li oxygen batteries, li oxygen cell
Zn-Air	zinc air battery, zinc air batteries, zinc air cell, zn air battery, zn air batteries, zn air cell
Supercaps	supercap, supercapacitor, capacitor
HER	her, hydrogen evolution reaction, hydrogen evolution, hydrogen catalysis, hydrogen production, h2 evolution reaction, h2 evolution, h2 catalysis, h2 production

Table SI 3 – API classification algorithm synonyms for category: production methods.

Field Word	Synonyms
Mechanical exfoliation	mechanical exfoliation, scotch tape method, ball mill, ball milled, ball milling
Sputtering	sputter, sputtering, sputtered
Liquid exfoliation	liquid exfoliation, liquid phase exfoliation, liquid assisted exfoliation, assisted liquid exfoliation, assisted liquid phase exfoliation, assisted exfoliation, sonication assisted exfoliation, sonication assisted liquid exfoliation, sonication assisted liquid phase exfoliation
Reductive chemistry (organo-alkali solvents + the metal ammonia method)	intercalation exfoliation, intercalation and exfoliation, intercalation assisted exfoliation, intercalation assisted liquid exfoliation, intercalation assisted liquid phase exfoliation, assisted intercalation exfoliation, assisted intercalation liquid exfoliation, assisted intercalation liquid phase exfoliation
Electrochemical	electrochemical exfoliation, electrochemical intercalation exfoliation, electrochemical assisted intercalation exfoliation
Hydrothermal	hydrothermal
Solvothermal	solvothermal
Solid State Reaction	solid state reaction, solid state synthesis, solid state production
CVD	cvd, chemical vapor deposition, chemical vapour deposition
Commercial MoS_2	commercial mos2, commercial molybdenum disulphide, commercial molybdenum disulfide, bulk mos2, bulk molybdenum disulphide, bulk molybdenum disulfide

Table SI 4 - API classification algorithm synonyms for category: characterization techniques. Synonyms in bold clash with larger synonyms from different sub-categories (italic), which contain much of the same terms.

Field Word	Synonyms
SEM	sem, scanning electron microscopy, scanning electron microscope, **electron microscopy**
TEM	**tem**, ***transmission electron microscopy***, **transmission electron microscope**, transmission imaging
HRTEM	hrtem, *hr tem*, *high resolution tem*, *high resolution transmission electron microscopy*, *high resolution transmission electron microscope*
HAADF-STEM	haadfstem, haadf stem, high angle annular dark field stem, *high angle annular dark field scanning transmission electron microscopy*, *high angle annular dark field scanning transmission electron microscope*
AFM	afm, atomic force microscopy, atomic force microscope
DLS	dls, dynamic light scattering, dynamic light scatter
Gas adsorption	n2 adsorption, nitrogen physisorption, n2 physisorption, gas physisorption, bet, brunauer emmet teller
XRD	xrd, xray diffraction, x ray diffraction
SAED	saed, selected area electron diffraction
XPS	xps, xray photoelectron spectroscopy, x ray photoelectron spectroscopy
EDS	eds, edx, energy dispersive spectroscopy, energy dispersive xray spectroscopy, energy dispersive x ray spectroscopy
TGA	tga, tg, thermogravimetric analysis, thermal analysis
DSC	dsc, differential scanning calorimetry
Raman	raman, raman spectroscopy
PL	photoluminescence
UV-vis	uvvis, uv vis, uv vi, uv visible, ultra violet visible spectroscopy
FTIR	ftir, ft ir, fourier transform infrared

Table SI 5: Manual and API classification method variance – sum of absolute difference from average count using Equation SI 1.

Figure	Description	Average (δ)	Variance (σ)
Fig. 1 A	API – Application 2011-2022	1475	8795
Fig. 1 B	Manual – Production Routes	8.3	100.7
Fig. 1 C	Manual – 1T Production Routes	2.4	28.7
Fig. SI 2	Manual – Application	12.5	82

Table SI 6: Median and literature MoS_2 XPS peak locations.

Peak	Peak Location (eV)	2H Peak Location (eV)	1T Peak Location (eV)	Source
**Li 1s region:**	50-62			
Li_2CO_3	55.2			(2)
**S 2p region:**	155-170			
S^{2} $2p_{3/2}$		161.95	161.45	Median
S^{2} $2p_{1/2}$		163.2	162.5	Median
**Mo 3d region:**	220-240			
S^{2} 2s		226.25		Median
Mo^{4+} $3d_{5/2}$		229.2	228.6	Median
Mo^{4+} $3d_{3/2}$		232.5	231.64	Median
Mo^{6+} $3d_{5/2}$	233.95			Median
Mo^{6+} $3d_{3/2}$	235.8			Median
**C 1s region:**	280-300			
C-C	284.6			Median
C-O	286.05			Median
C-O-Mo	286.2			(3)
C=O	288.1			Median
C=N	288.4			(4)
O-C-O	288.9			(5)
C (pi-pi*)	290.1			(6)
**N 1s region:**				
C-N	395.2			(4)
C=N	397.9			(4)
N-Mo	401.7			(4)
**O 1s region:**	524-544			
C=O	531.2			(5)
C-O-Mo	532.4			(5)
C-OH	533.3			(5)

Table SI 7: MoS_2 and carbon Raman modes. Median analysis and literature reporting.

**Peak (cm^{-1})**	**Mode**	**Phase/Material**	**Source**
32	E_{2g}^2	2H MoS2	(7,8)
147	J_1	1T MoS2	Median
190	Z_1	2H/1T MoS2	Median
222	J_2	1T MoS2	Median
281	E_{1g}	2H MoS2	Median
333	J_3	1T MoS2	Median
379	E_{2g}^1	Top-down precursor/2H/Heterogeneous 1T	Median
405	A_{1g}	Top-down precursor/2H/Heterogeneous 1T	Median
450	B_{2g}^1	Top-down precursor/2H	(7)
1346	D	Graphite	Median
1587	G	Graphite	Median
~1620	D’	Graphite	(9)
~2450	D + D’’	Graphite	(9)
~2700	2D or G’	Graphite	(9)
~2950	D’’	Graphite	(9)
~3250	2D’	Graphite	(9)

References:

1. Shi, S.; Sun, Z.; Hu, Y. H. Synthesis, stabilization and applications of 2-dimensional 1T metallic MoS2. Journal of Material Chemistry A **2018**, 6, 23932–23977

2. Ahmadiparidari, A.; Warburton, R. E.; Majidi, L.; Asadi, M.; Chamaani, A.; Jokisaari, J. R.; Rastegar, S.; Hemmat, Z.; Sayahpour, B.; Assary, R. S.; Narayanan, B.; Abbasi, P.; Redfern, P. C.; Ngo, A.; Vörös, M.; Greeley, J.; Kile, R.; Curtiss, L. A.; Salehi-Khojin, A. A Long-Cycle-Life Lithium–CO2 Battery with Carbon Neutrality. Advanced Materials **2019**, 31, 1–7

3. Bai, J.; Zhao, B.; Zhou, J.; Si, J.; Fang, Z.; Li, K.; Ma, H.; Dai, J.; Zhu, X.; Sun, Y. Glucose-Induced Synthesis of 1T-MoS2/C Hybrid for High-Rate Lithium-Ion Batteries. Small **2019**, 15, 1–11

4. Wang, T.; Sun, C.; Yang, M.; Zhao, G.; Wang, S.; Ma, F.; Zhang, L.; Shao, Y.; Wu, Y.; Huang, B.; Hao, X. Phase-transformation engineering in MoS2 on carbon cloth as flexible binder-free anode for enhancing lithium storage. Journal of Alloys and Compounds **2017**, 716, 112–118

5. Teng, Y.; Zhao, H.; Zhang, Z.; Li, Z.; Xia, Q.; Zhang, Y.; Zhao, L.; Di, X.; Du, Z.; Lv, P.; Śweirczek, K. MoS2 Nanosheets Vertically Grown on Graphene Sheets for Lithium-Ion Battery Anodes. ACS Nano **2016**, 10, 8526–8535

6. Ma, L.; Zhao, B.; Wang, X.; Yang, J.; Zhang, X.; Zhou, Y.; Chen, J. MoS2 Nanosheets Vertically Grown on Carbonized Corn Stalks as Lithium-Ion Battery Anode. ACS Applied Materials & Interfaces **2018**, 10, 22067–22073

7. Placidi, M.; Dimitrievska, M.; Izquierdo-Roca, V.; Fontané, X.; Castellanos-Gomez, A.; Pérez-Tomás, A.; Mestres, N.; Espindola-Rodriguez, M.; López-Marino, S.; Neischitzer, M. Multiwavelength excitation Raman Scattering Analysis of bulk and 2 dimensional MoS2: Vibrational properties of atomically thin MoS2 layers. 2D Materials **2015**, 2

8. Li, H.; Zhang, Q.; Yap, C. C. R.; Tay, B. K.; Edwin, T. H. T.; Olivier, A.; Baillargeat, D. From bulk to monolayer MoS2: Evolution of Raman scattering. Adv Funct Mater **2012**, 22, 1385–1390

9. Ferrari, A. C.; Basko, D. M. Raman spectroscopy as a versatile tool for studying the properties of graphene. Nature Nanotechnology **2013**, 8, 235–246
